# Nanoparticle‐mediated genome editing in single‐cell embryos via peptide nucleic acids

**DOI:** 10.1002/btm2.10458

**Published:** 2022-12-02

**Authors:** Rachael Putman, Adele S. Ricciardi, Kelly E. W. Carufe, Elias Quijano, Raman Bahal, Peter M. Glazer, W. Mark Saltzman

**Affiliations:** ^1^ Department of Biomedical Engineering Yale University New Haven Connecticut USA; ^2^ Department of Therapeutic Radiology Yale University New Haven Connecticut USA; ^3^ Department of Genetics Yale University New Haven Connecticut USA; ^4^ Department of Cellular & Molecular Physiology Yale University New Haven Connecticut USA; ^5^ Department of Chemical & Environmental Engineering Yale University New Haven Connecticut USA; ^6^ Duke University School of Medicine Durham North Carolina USA; ^7^ Department of Surgery University of Pennsylvania Health Systems Philadelphia Pennsylvania USA; ^8^ Department of Pharmaceutical Sciences University of Connecticut Storrs Connecticut USA

**Keywords:** embryo editing, gene editing, nanoparticles, nucleic acid chemistry, peptide nucleic acids, PNA, prenatal gene therapy

## Abstract

Through preimplantation genetic diagnosis, genetic diseases can be detected during the early stages of embryogenesis, but effective treatments for many of these disorders are lacking. Gene editing could allow for correction of the underlying mutation during embryogenesis to prevent disease pathogenesis or even provide a cure. Here, we demonstrate that administration of peptide nucleic acids and single‐stranded donor DNA oligonucleotides encapsulated in poly(lactic‐*co*‐glycolic acid) (PLGA) nanoparticles to single‐cell embryos allows for editing of an eGFP‐beta globin fusion transgene. Blastocysts from treated embryos exhibit high levels of editing (~94%), normal physiological development, normal morphology, and no detected off‐target genomic effects. Treated embryos reimplanted to surrogate moms show normal growth without gross developmental abnormalities and with no identified off‐target effects. Mice from reimplanted embryos consistently show editing, characterized by mosaicism across multiple organs with some organ biopsies showing up to 100% editing. This proof‐of‐concept work demonstrates for the first time the use of peptide nucleic acid (PNA)/DNA nanoparticles as a means to achieve embryonic gene editing.

## 1 INTRODUCTION

Genome editing has the potential to treat numerous genetic disorders. Embryonic gene editing may have advantages over editing in adults due to the smaller number of cells, which are rapidly dividing to form the rest of the organism. Changes made in these stem cells may be durable and passed onto daughter cells, potentially allowing for high editing rates that may be maintained throughout adulthood. Embryonic gene editing could be used for many applications, such as the creation of genetic mouse models and improving the output of livestock products, and it may eventually have clinical utility during in vitro fertilization (IVF) for the treatment or prevention of human disease. Although preimplantation genetic diagnosis during IVF allows for selection of healthy embryos, there may be clinical scenarios where a higher number of potentially viable embryos for transfer may be desired. For example, due to higher numbers of affected embryos in X‐linked and autosomal dominant genetic disorders, or due to advancing age of the oocytes of the biological mother and a decreased chance of a successful IVF cycle, families may wish for alternative treatments that could increase the number of healthy embryos available to improve the chance of the birth of a healthy child. In addition to couples that are known carriers of mutations, this approach may also be particularly applicable to couples undergoing IVF who are both homozygous for a monogenic disease.

Traditional embryonic gene editing approaches involve pronuclear microinjection or electroporation of engineered nucleases, such as CRISPR/Cas9. While capable of achieving high levels of gene editing, pronuclear microinjection is labor intensive, requires specially trained personnel, and both pronuclear microinjection and electroporation methods can result in significant embryo loss.^1^, ^2^, ^3^, ^4^, ^5^ Recombinant AAV vectors have been shown to deliver CRISPR/Cas9 reagents through the zona pellucida (ZP) of mice without the need for electroporation or microinjection with high levels of gene editing without significant embryo loss.^6^ However, integration of AAV into the genome can occur,^6^, ^7^ which may increase the possibility of off‐target effects and limit potential applications for human embryonic gene editing.

Our approach uses nanoparticles (NPs) fabricated from a biodegradable polymer, poly(lactic‐*co*‐glycolic acid) (PLGA), which has been used in humans for over 40 years and is well known to be safe. To achieve gene correction, two active agents are loaded into the NPs: a triplex‐forming peptide nucleic acid (PNA) that induces recombination at a specific, targeted site on a chromosome and a short donor DNA molecule that contains the desired gene sequence. Once administered, PLGA nanoparticles are taken up by cells where they degrade, releasing the loaded PNA and DNA. Once inside a cell, the engineered PNA binds to a specific genomic target site, forming a triplex, which induces DNA repair mechanisms that stimulate the recombination of the short, single‐stranded donor DNA molecule containing the correct sequence, resulting in site‐specific gene editing. This approach to gene editing has been previously shown to be capable of achieving site‐specific gene editing with extremely low levels of off‐target sequence modifications and to be safe in both adult and fetal animals,^8^, ^9^, ^10^, ^11^, ^12^, ^13^ which may be an advantage for PLGA PNA/DNA‐loaded NPs over nuclease‐based gene editing techniques such as zinc‐finger nucleases, CRISPR/Cas9, and TALENs technologies, whose potential off‐target, cytotoxic, and/or immunogenic effects may hinder in vivo gene editing applications.^14^, ^15^

In prior work, we demonstrated the feasibility and safety of PNA/DNA NP‐mediated gene editing in adult mice and fetal mice. Here, we sought to determine the feasibility, safety, and efficacy of embryonic gene editing mediated by treatment ex vivo with PNA/DNA‐loaded NPs. We find that NP treatment at the single‐cell zygote stage allows for normal physiological cell division with no significant cytotoxicity, and treated embryos are edited at substantial rates with no detected off‐target effects above background by deep sequencing. Reimplanted murine embryos are capable of normal growth and differentiation with no observed developmental abnormalities. Reimplanted eGFP embryos also exhibit high levels of gene editing, albeit with significant mosaicism, and exhibit phenotypic expression of corrected eGFP, establishing the potential for embryonic gene editing with PNA/DNA NPs.

## 2 RESULTS

### 2.1 Biodistribution and safety of nanoparticle treatment of mouse embryos

Mammalian oocytes are protected by a glycoprotein coating called the ZP. The mammalian ZP is an extremely porous coat that can be penetrated by large macromolecules, such as antibodies and enzymes, as well as by small viruses including AAV and BHV1.^6^, ^16^, ^17^, ^18^ Most viruses typically range from 20 to 200 nm; AAV is about 20 nm^17^ and BHV1 is about 180–200 nm.^19^ The PLGA NPs used for delivery, which were created with a double‐emulsion solvent evaporation technique modified to encapsulate PNA and DNA oligomers as previously described,^8^, ^20^ had an average hydrodynamic diameter around 250 nm; additional characterization data are included in Table S1 and imaging of NPs is included in Figure S1.

**FIGURE 1:**
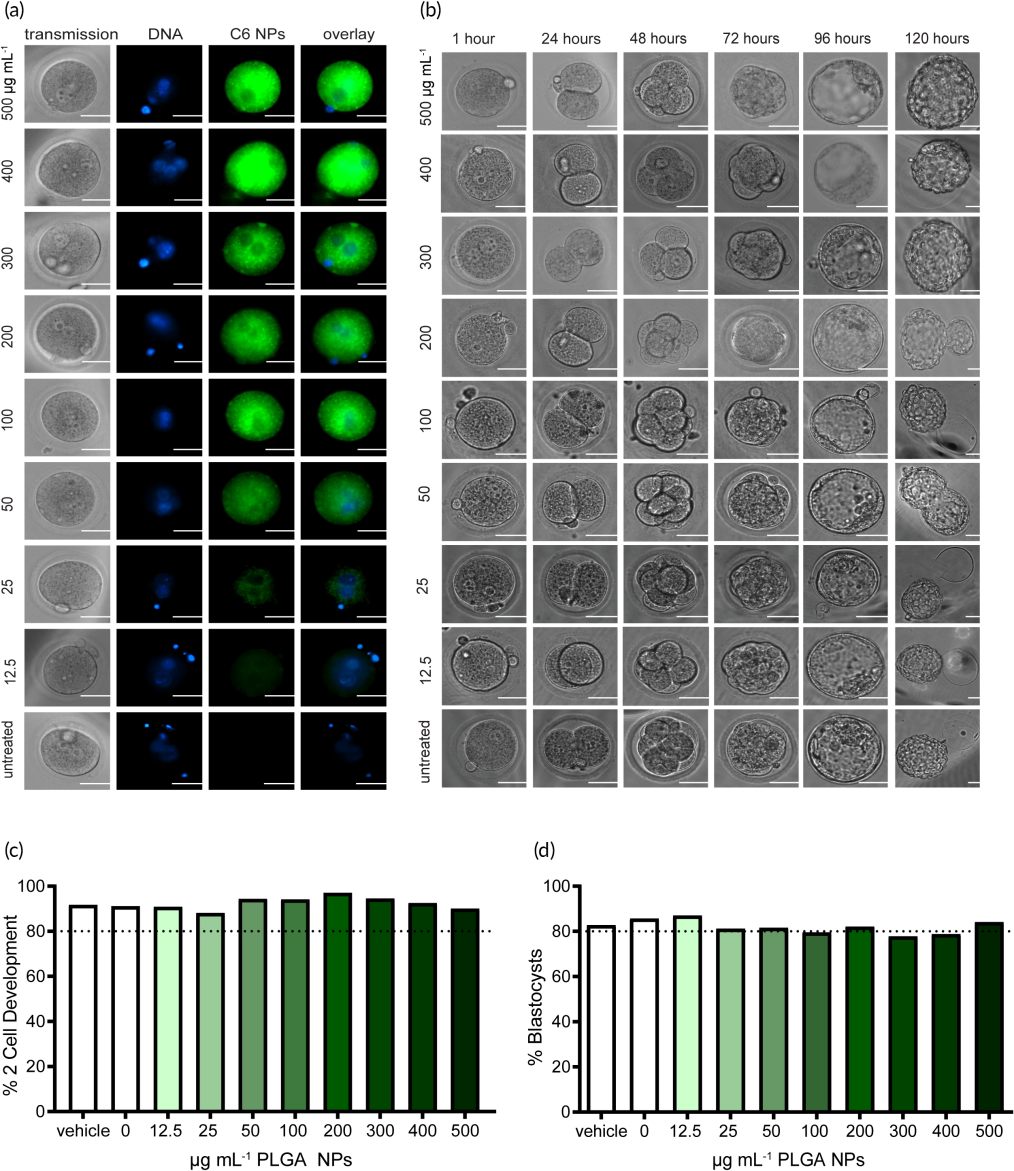
Delivery of poly(lactic‐*co*‐glycolic acid) (PLGA) nanoparticles (NPs) to single‐cell embryos. (a) Uptake of coumarin‐6 dye loaded PLGA NPs 1 h after addition to medium containing single‐cell embryos. Nuclei were stained with a live‐cell nuclear stain (Hoescht dye) (blue), scale bars = 50 μm. (b) Representative image of embryo morphology and division for various treatment doses over 5 days, scale bars = 50 μm. (c, d) Development to the two‐cell (c) and blastocyst stage (d) of treated and untreated groups. Nanoparticle dose in μg ml^−1^ is indicated on graph; vehicle indicates NPs at a concentration of 500 μg ml^−1^ that contained no cargo. The horizontal dotted line at 80% indicates the expected untreated embryo survival based on culture methods used. Survival percentages and number of embryos per group are included in Table S2 (two‐cell stage) and Table S3 (blastocyst stage).

Given the size of the PLGA NPs used, to investigate whether the NPs were capable of penetrating the ZP of fertilized mouse oocytes, NPs loaded with a fluorescent dye (coumarin‐6, C6) were added to medium containing single‐cell embryos. After incubation for 1 h, the embryos were washed and then imaged for the presence of fluorescence associated with the NPs. All tested doses (12.5 –500 μg ml^−1^) resulted in widespread NP accumulation throughout the embryo, indicating that the 250 nm PLGA NPs can traverse the ZP (Figure 1a). Based on comparison to the brightfield images, C6‐NP signal was seen within the cell but not seen in the perivitelline space, the zona pellucida (ZP), or extracellular medium. In addition, we observed nuclear clearing within the pronuclei, which would not occur if the signal was not intracellular and intranuclear. Based on these findings, we conclude that the NPs are intracellular (Figure 1a) and that intracellular accumulation was dose dependent (Figure 1a).

Although NPs can penetrate the ZP, to achieve gene editing, PNA and donor DNA reagents must be released from the NP and then reach the nucleus. One potential advantage of embryo editing with PNA/DNA NPs is the elimination of the technically challenging pronuclear microinjection step that is used for gene editing with CRISPR/Cas9, but this requires that NPs provide nuclear delivery of the reagents. To determine whether NPs were reaching the nuclei of treated embryos, cells were stained with Hoechst, a nuclear stain that allows for live cell imaging, and imaged 1 h after fluorescent NP delivery. There was overlap between fluorescence from C6‐loaded NPs and the Hoechst‐stained embryo pronuclei (Figure 1a), indicating nuclear delivery. However, there was some nuclear clearing observed at the 1 h time‐point, representing a region of the cell that contains fewer fluorescent NPs within the region of the nuclear staining (Figure 1a), indicating less delivery to the nucleus relative to the intracellular space. However, at the beginning of mitosis, the nuclear envelope disassembles, allowing for mixing of the nuclear and cytoplasmic contents. During telophase, the nuclear envelope reforms around the condensed chromosomes at either pole of the cell. Nuclear envelope breakdown and reassembly could allow for the relocalization of NPs that were initially in the cytoplasm to the nucleus, and this could allow for high levels of editing even if NPs are not initially in high concentrations within the nucleus.

The safety of this approach in vitro was assessed. Two measures of embryo health in culture are development to the two‐cell stage and blastocyst hatching from the ZP.^21^ During the early stages of embryo development, the ZP prevents the embryo from implanting into the oviduct of a mouse, or the Fallopian tube of a human. Once the embryo traverses the oviduct and reaches the uterus, a healthily dividing embryo should hatch from the ZP to allow for implantation into the uterine wall. We observe that the NP‐treated embryos hatch from the ZP in culture (Figure 1b) and that NP treatment does not negatively impact the percentage of embryos reaching the two‐cell stage or the blastocyst stage for any of the NP doses tested (Figure 1c,d). Survival percentages and number of embryos per group are included in Table S2 (two‐cell stage) and Table S3 (blastocyst stage). Untreated embryos are expected to have greater than 80% development to the two‐cell and blastocyst stage based upon the medium and culture methods used; treatment with NPs does not appear to impair cellular division or to be otherwise cytotoxic.

### 2.2 Delivery of PNAs into embryos

**FIGURE 2:**
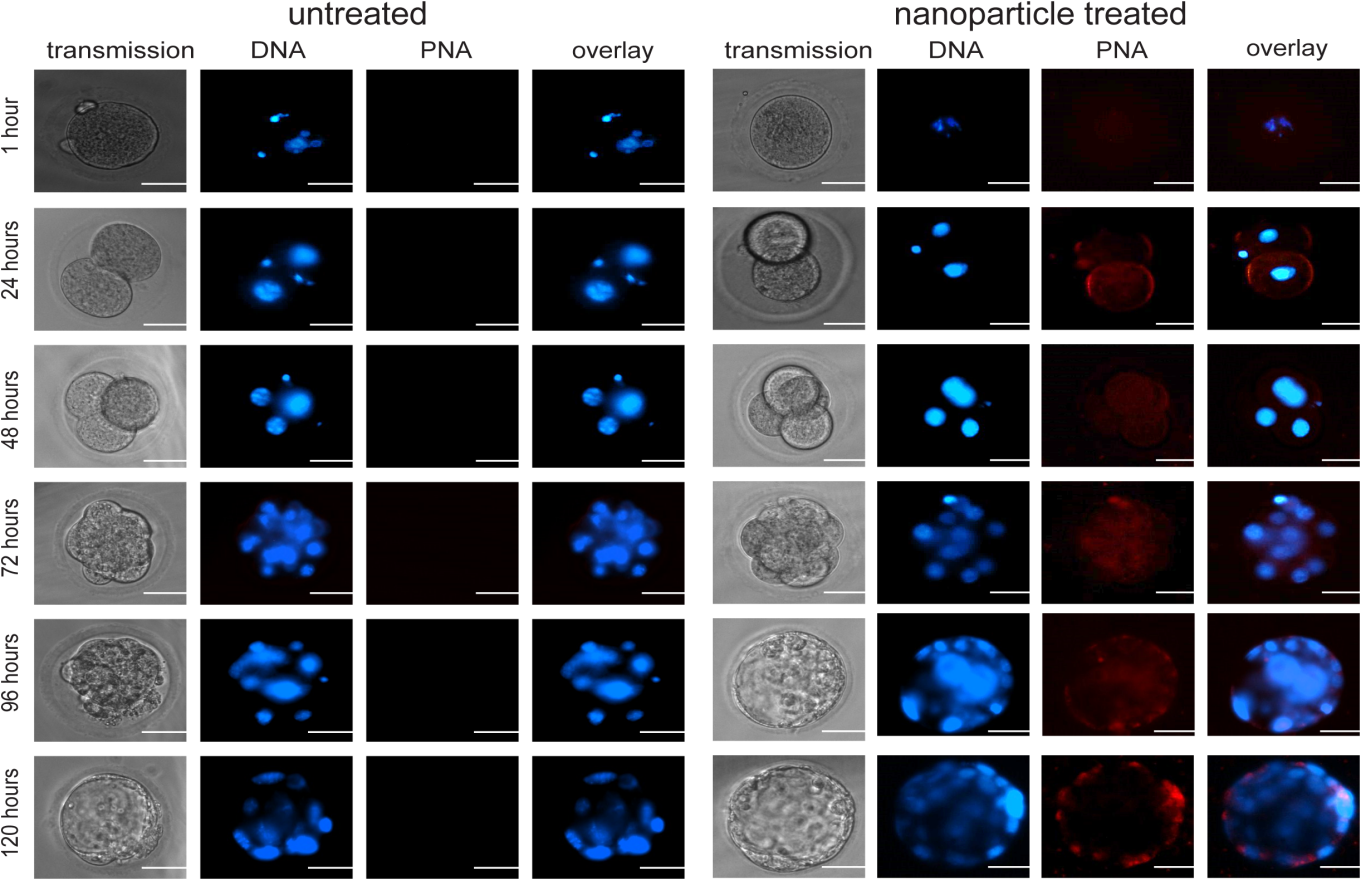
Delivery of TAMRA‐PNA by poly(lactic‐*co*‐glycolic acid) (PLGA) nanoparticles (NPs) to single‐cell embryos in culture. Representative images for nanoparticle mediated delivery of TAMRA‐labeled PNA NPs (red) to embryos in culture. Embryos were monitored for 5 days. Nuclei were stained with Hoescht dye (blue), scale bars = 50 μm.

To examine the delivery of PNAs to embryos by NP treatment, PLGA NPs containing PNA fluorescently labeled with tetramethylrhodamine (TAMRA) were fabricated. Note that the labeled‐PNA containing NPs are much dimmer on imaging than the C6 dye NPs, since the concentration of C6 in the NPs (0.3%) is much higher than the concentration of the labeled PNAs in the NPs (~0.02%). Embryos were treated with TAMRA‐labeled PNA for 1 h then maintained in culture for 5 days, with daily imaging (Figure 2). Fluorophore signal correlates with PNA concentration. After 1 h, there is greater than background fluorescence present within the embryo; however, the signal is very faint. By the two‐cell stage, PNA is widely distributed within the embryo, including the nuclei as shown by overlap of the Hoechst nuclear stain and the red TAMRA signal. The PNA signal persists throughout the cells for at least 5 days. The PNAs in the representative image at 120 h appear at the periphery since cells at this stage are located around the outside of the blastocyst. These cells include trophoblast cells that will go on to form the extraembryonic membranes and the placenta, and the inner cell mass, which will go on to form the fetus. The center of the blastocyst is the cell‐free fluid‐filled blastocoel cavity, which assists in generating sufficient pressure for zona hatching. In addition, the images indicate that treatment at the one‐cell stage with PNA and DNA containing NPs did not impede further cell division or development into blastocysts.

### 2.3 Analysis of editing in vitro at the blastocyst stage

**FIGURE 3:**
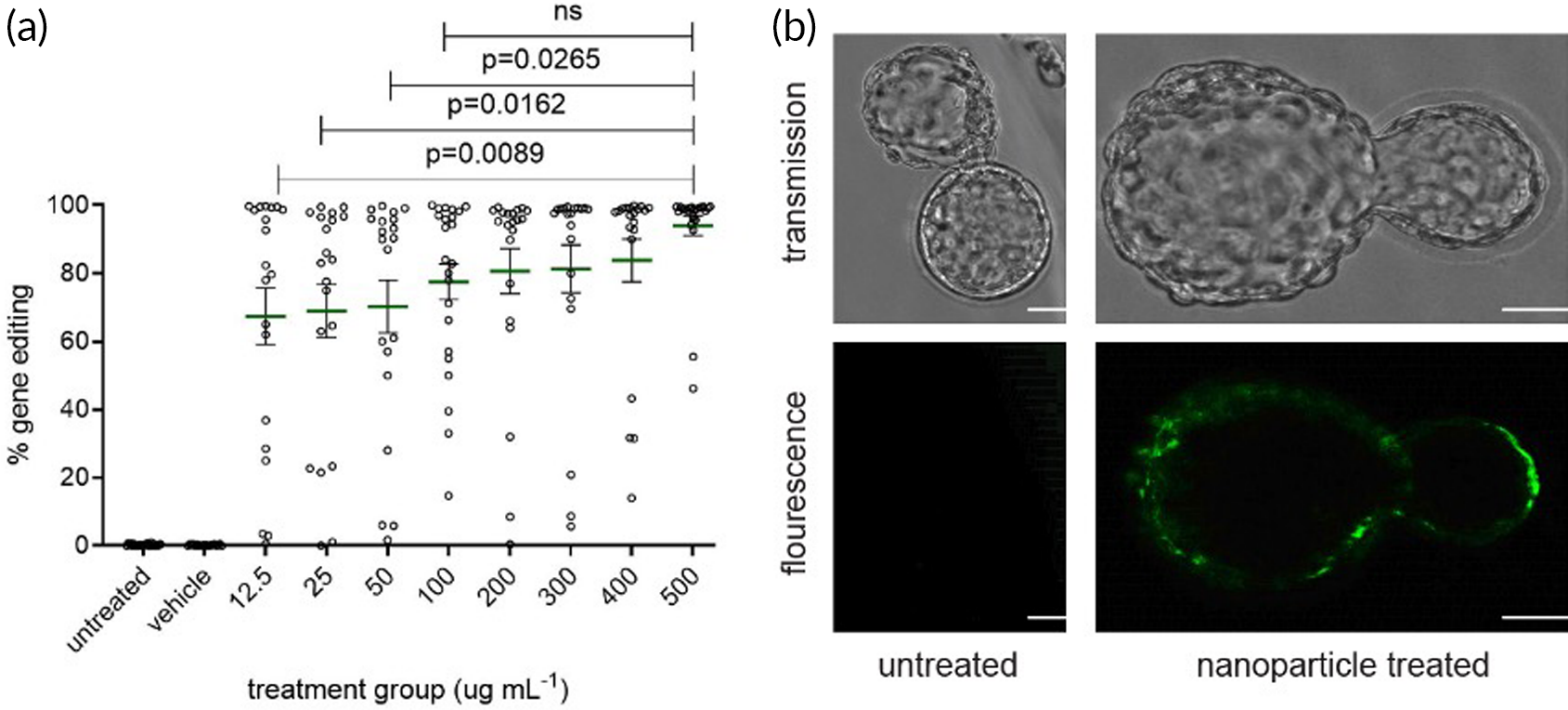
Gene editing quantified by droplet digital PCR (ddPCR) and visualized by fluorescence microscopy in 654‐eGFP blastocysts following treatment of single‐cell embryos with PNA nanoparticles. (a) Gene editing in blastocysts after PNA/DNA NP treatment by ddPCR analysis with mean ± s.e.m. Significance by one‐way ANOVA analysis for various treatment groups compared with 500 μg ml^−1^ NP group is shown. Groups above 100 μg ml^−1^ NP dose were not significantly different, marked as ns. ANOVA of significance and number of samples for different treatment groups compared to untreated control is included in Table S4. Vehicle group is 500 μg ml^−1^ poly(lactic‐*co*‐glycolic acid) (PLGA) NPs containing no cargo. (b) Representative images for eGFP fluorescence in a treated and untreated e5.5 embryo, scale bars = 50 μm

To allow for analysis of gene editing both at the genotype and phenotype level, we used embryos derived from a transgenic reporter mouse (designated 654‐eGFP) containing a GFP‐beta globin fusion transgene with a IVS2‐654 (C to T) mutation in the beta globin‐derived intron causing an aberrantly spliced mRNA; correction by gene editing results in expression of a functional GFP mRNA transcript.^22^ (Figure S2). To analyze levels of editing achieved in vitro, single‐cell fertilized embryos were harvested from homozygous 654‐eGFP mice and PLGA NPs containing PNA and donor DNA designed to correct the IVS2‐654 mutation^2^ were pipetted into medium. After 1 h of treatment, embryos were washed and transferred to fresh medium. Editing was evaluated 5 days post‐NP treatment at the blastocyst stage. Genomic DNA (gDNA) was isolated from individual blastocysts when an embryo contains approximately 100 cells. Since these 654‐eGFP mice each have two transgenes, this results in approximately 200 detectable alleles. To allow quantification of editing, we performed whole genome amplification on the gDNA samples from the blastocysts and then the efficiency of gene editing was measured by droplet digital PCR (ddPCR) (Figure 3a). Controls were performed to confirm that the whole genome amplification step does not skew the results of the ddPCR assay (Figure S3).

A range of NP doses (12.5–500 μg ml^−1^) was investigated. The mean level of editing at the blastocyst stage increased with increasing NP dosage (Figure 3a), consistent with higher delivery of PNA leading to more potential binding and editing events in treated embryos. At all NP doses tested, embryonic gene editing was statistically significant (*p* < 0.0001) when analyzed by a one‐way ANOVA test comparing each treatment group to untreated controls (Table S4) and even the lowest tested dose of 12.5 μg ml^−1^ resulted in an average level of editing of 67% (Figure 3a). At the highest dose tested of 500 μg ml^−1^, a 94% average editing of each blastocyst was achieved; 19 of the 21 blastocysts investigated at the 500 μg ml^−1^ dose exhibited very high levels of editing approaching 100% and 2 embryos exhibited editing around 50% (Figure 3a). Deep sequencing was also performed on blastocysts to confirm on target editing (Table S5).

Additionally, eGFP fluorescence was observed in multiple treated embryos at the blastocyst stage (e4.5/e5.5) (Figure 3b). The eGFP signal appears localized around the outside of the blastocyst, which is consistent with the localization of cells at the periphery of the blastocyst, surrounding the blastocoel cavity. No eGFP fluorescence was observed in untreated embryos (Figure 3b). Seventeen of the 21 analyzed blastocysts (81%) had eGFP fluorescence by a visual assessment of microscopy images. This is lower than what would be expected based on the ddPCR results of the 500 μg ml^−1^ dose, however, embryos may be edited but not expressing eGFP at high enough levels that it was able to be imaged on the microscope as above background fluorescence.

### 2.4 Safety of embryonic nanoparticle treatment in reimplanted mice

Single‐cell fertilized embryos containing two copies of the 654‐eGFP transgene were harvested. Embryos were treated for 1 h with PLGA NPs containing the PNA and donor DNA designed to correct the IVS2‐654 mutation, as previously described,^8^ at a concentration of 500 μg ml^−1^ in the medium and then cultured for 1 day before transfer to surrogate mothers at the two‐cell stage.

The 654‐eGFP embryos treated with PNA/DNA NPs and reimplanted at the two‐cell stage had a development rate after reimplantation similar to that expected for untreated embryos (Table 1); untreated reimplanted embryos yield pups at a rate of approximately 50%.^23^, ^24^ In our studies, 54 out of 123 NP‐treated embryos (44%) successfully implanted and developed to birth compared to 39 of 83 untreated embryos (47%). Pronuclear microinjection of CRISPR/Cas9 results in a reported development rate after reimplantation at the single‐cell stage of approximately 30%,^25^ constituting a significant embryo loss. PNA/DNA NP treatment may therefore represent a gentler method for achieving site‐specific embryonic gene editing.

**TABLE 1:** Reimplantation rates for PNA/DNA nanoparticle treatment

Treatment	Development at reimplantation	Embryos reimplanted	Pups	Percent survival (%)
Untreated	Single cell	44	24	54.5
Untreated	Two cell	83	39	47.0
PNA/DNA	Single cell	139	64	46.0
PNA/DNA	Two cell	54	123	43.9

*Note*: Pups include both healthy fetuses harvested at e17.5 and pups born and survived to weaning.

PNA/DNA NP treatment did not interfere with growth and development of the treated mice. All pregnancies resulted in fetuses that appeared healthy and exhibited normal morphology. E17.5 fetuses and postnatal pups exhibited no visible defects and displayed normal development (Figure S4). Additionally, mice derived from treated embryos had normal growth when compared to mice from litters with a similar number of pups, as monitored for 30 days after birth (Figure S4). Mortality of untreated (*n* = 30) and treated reimplanted mice (*n* = 32 mice reimplanted at two‐cell stage and *n* = 32 reimplanted at single‐cell stage following embryo treatment) that survived to weaning was similar at 3 months, with no deaths in the experimental or control group during this time. Additionally, no tumors were found in the treated or untreated reimplanted mice (*n* = 30 untreated, *n* = 32 treated and reimplanted at the single‐cell stage, *n* = 32 treated and reimplanted at the two‐cell stage). Behavior of both untreated and treated reimplanted mice during this period was grossly normal. While this does not fully establish the safety of this approach, it does indicate that PNA/DNA NP treatment is not overtly toxic to the treated mice and is compatible with apparently normal growth and development.

### 2.5 Analysis of editing following reimplantation and development in utero

**FIGURE 4:**
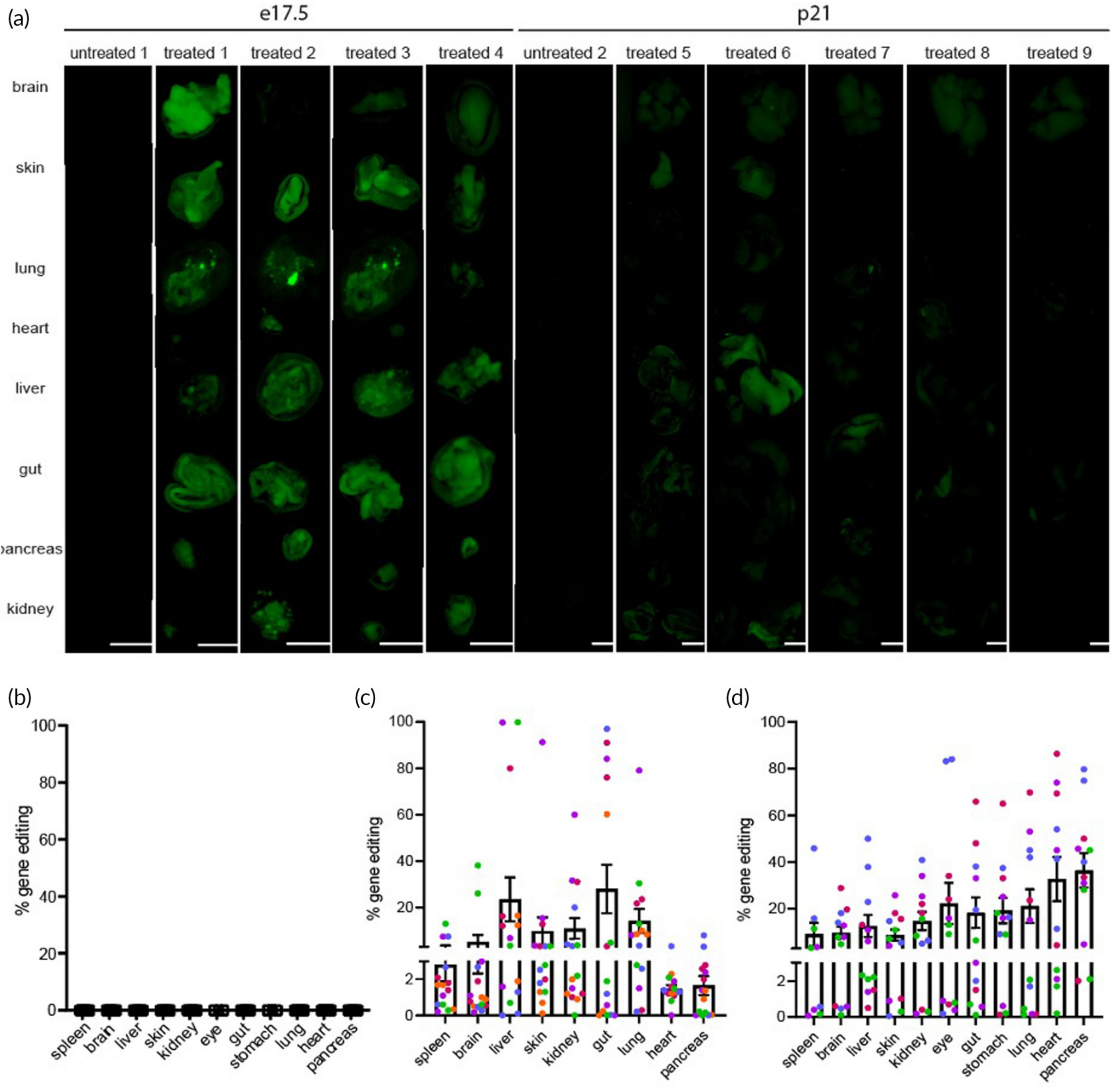
Phenotypic evidence of gene editing via green fluorescence and quantitation of editing by droplet digital PCR (ddPCR) in mice after reimplantations of treated 654‐eGFP embryos at the two‐cell stage. (a) eGFP fluorescent signal in tissues of untreated and treated mice. Untreated mouse 1 and treated mice 1–4 were analyzed at embryonic day of life e17.5; untreated mouse 2 and treated mice 5–9 were analyzed at post‐natal day of life p21. (b–d) ddPCR analysis of editing for organs of (b) untreated mice, (c) treated mice at e17.5, and (d) treated mice at p21. Percentages from biopsies are shown with mean ± s.e.m., scale bars = 2.5 mm. Three biopsies were taken from each tissue for each mouse. Untreated mouse 1 and 2 were plotted in (b), treated mice 1–4 (harvested at e17.5) were plotted in (c) and treated mice 4–9 (harvested at p21) were plotted in (d). Each mouse was assigned its own color in the plots. For (b), treated mouse 1 is assigned pink, treated mouse 2 is assigned blue, treated mouse 3 is assigned purple, and treated mouse 4 is assigned green. For (c), treated mouse 5 is assigned purple, treated mouse 6 is assigned pink, treated mouse 7 is assigned green, treated mouse 8 is assigned orange, and treated mouse 9 is assigned blue.

654‐eGFP embryos reimplanted at the two‐cell stage were analyzed for phenotypic evidence of editing (based on green fluorescence) as fetuses on embryonic day e17.5 or as pups on post‐natal day p21. Multiple pups from NP‐treated embryos exhibited robust fluorescence in numerous organs as visualized on a Leica stereomicroscope (Figure 4a). The tissues had varying levels of autofluorescence, so each tissue type was imaged at settings where untreated pups showed no fluorescence in that organ. Additional untreated control images are shown in Figure S5 and additional images taken using the IVIS Spectrum in vivo imaging system are shown in Figures S6 and S7. Treated pups were seen to have varying degrees of mosaicism as evidenced by heterogeneity in the GFP fluorescence signal between or within organs (Figure 4a).

To further evaluate the level of editing at the genomic level, we performed ddPCR on gDNA samples taken from the various tissues of the e17.5 (Figure 4c) or p21 pups (Figure 4d). Multiple biopsy samples were taken from each tissue and analyzed separately for degree of editing by ddPCR, which showed different levels of editing within the same organ of each mouse, indicating mosaicism (Figure 4b,c). To confirm on‐target editing, deep sequencing was also performed on treated tissues (Table S5).

Most of the eGFP pups that resulted from embryos reimplanted at the two‐cell stage showed some degree of editing by ddPCR and exhibited corresponding fluorescence in multiple organs. Based on ddPCR, in pups analyzed at e17.5, 12 of 13 pups (92%) had at least some organs with measurable editing, and 5 of 13 pups had some level of editing in all evaluated organs (35.7%). In pups analyzed at p21, 10 of 11 pups (91%) had at least some organs with measurable editing, and 4 of 11 pups had some level of editing in all their organs (36%).

Of note, mice reimplanted at the two‐cell stage and analyzed at p21 exhibit significantly decreased fluorescence intensity relative to the e17.5 mice (Figure 4a), but similar levels of editing overall (Figure 4c,d), albeit with both e17.5 and p21 pups characterized by the presence of significant mosaicism. This was particularly evident in the IVIS images (Figures S6 and S7). This may be partially due to absorption spectrum of eGFP, with excitation at 488 nm and detection at 510 nm, as hemoglobin effectively absorbs light below 600 nm and thus results in some expected quenching of eGFP fluorescence.^26^ This effect may be less pronounced in the e17.5 mice as fetal and neonatal mice typically have lower numbers of RBCs than adult mice,^27^ thus potentially leading to less quenching of the fluorescent signal. Additionally, GFP can exhibit cytotoxicity to multiple organ systems,^28^ which may result in downregulation of expression and explain some of the decreased fluorescence seen in the adult mice.

Mice treated as embryos and reimplanted at the two‐cell stage were also characterized by significant mosaicism both between and within multiple organs (Figure 4). Reimplantation studies were also performed at the single‐cell stage to investigate whether this would affect the degree of mosaicism. Embryos were cultured for 1 h with NPs and then immediately reimplanted into surrogate mothers. Some editing was achieved with single‐cell reimplantations; however, editing and organ fluorescence were lower than in the two‐cell reimplanted pups (Figure S8), and mice were still characterized by significant mosaicism. Nine of 11 pups analyzed at e17.5 had at least one or more organs with some editing (82%), but no pups (0%) had editing in all of their organs, compared to 9 of 24 pups (38%) that had editing in all their organs with two‐cell reimplantations, indicating that the two‐cell reimplantations gave improved editing results. Reimplantations were not performed at later developmental stages due to decreased expected survival from standard surgical transfer techniques in mice.^29^

Analysis for statistical significance of editing by *t*‐test was performed with most organs of mice treated as embryos and reimplanted at the two‐cell stage showing significant editing. Most organs of mice treated as embryos and reimplanted at the single‐cell stage did not meet cutoffs for statistical significance. *p* values for each organ are included in Table S6.

### 2.6 Off‐target analysis

Whole genome sequencing was performed on reimplanted pups to ascertain whether there are any off‐target effects of the PNA/DNA NP treatment at any predicted or unexpected sites in the genome (Table 2). Trio sequencing was performed, where pups and both biological parents were sequenced to determine whether there are any mutation levels in the pups above background. Treated mice that underwent whole genome sequencing were mice 7, 8, and 9 from Figure 4a. As a control, an untreated pup and both biological parents were sequenced to establish a baseline for mutation levels caused naturally through genetic recombination. gDNA for whole genome sequencing was taken from mouse livers. All three treated mice had some degree of mosaicism present in livers, with some alleles edited and some unedited (Figure 4c). Based on total sampled alleles from the liver, treated mouse 7 had 31.9% editing, treated mouse 8 had 16.5% editing, and treated mouse 9 had 2.8% editing in the liver. Untreated and treated mice had similar levels of total identified variants with no significant differences between the groups (*p* = 0.1877) evaluated by one‐way ANOVA. Parents of treated and untreated mice were sequenced as well, with no significant differences in level of variation between parents or offspring. Variations present in either biological parents were filtered out of the total identified variations since variations present in either parent could be inherited and thus would not be candidates for de novo mutations. Untreated and treated mice had similar levels of variants after filtering (*p* = 0.1499), evaluated by one‐way ANOVA (Table 2).

**TABLE 2:** Results of whole genome sequencing of three littermates and their biological parents

Mouse number	Total variants	Average total variants of group	Filtered variants	Average filtered variants of group
Untreated 1	1422	1942	575	781
Untreated 2	2689		430	
Untreated 3	1714		1338	
Parent of untreated‐1	1124	1189	N/A	N/A
Parent of untreated‐2	1253		N/A	
Treated 7	1155	1033	291	227
Treated 8	663		43	
Treated 9	1281		346	
Parent of treated‐1	1367	1318	N/A	N/A
Parent of treated‐2	1268		N/A	

*Note*: The total number of variants, average total number of variants per group, number of variants after filtering out variants present in either biological parent and average number of filtered variants per group are reported above. Mice had similar numbers of total identified variants with nonsignificant differences between groups (*p* = 0.1877) and similar numbers of variants after filtering (*p* = 0.1499) by one‐way ANOVA.

Deep sequencing was also performed to analyze off‐target effects in treated mice at seven sites in the genome with partial homology to the PNA binding site in both blastocysts and reimplanted pups. Four treated tissues from different mice were analyzed for each site of partial homology (Table 3). Two untreated mice were also analyzed for each site; no modifications were found in untreated controls nor the treated samples. Three treated blastocysts were also analyzed for each site with partial homology (Table 4). Each blastocyst has approximately 100 cells; therefore, homozygous mice are expected to have approximately 200 alleles analyzed. No modifications were found in the treated samples (Table 4). One untreated blastocyst was also analyzed as a control; no modifications were found.

**TABLE 3:** Seven gene loci in the mouse genome with partial homology to the 18 bp γPNA target site in beta‐globin intron 2 were previously identified,^2^ with the sequences as indicated

Site name	Sequences of partial homology (5′–3′)	Size of region sequenced	Analyzed amplicons	Number modified	Frequency (%)
Off Target 1	AGCCCTGAAAGAAAGAGA	111	40,000	0	<0.2
Off Target 2	GAACCTGAAAGAAAGAGA	101	40,000	0	<0.2
Off Target 3	CACCCTGAAAGAAAGAAG	115	40,000	0	<0.2
Off Target 4	AAGCCTGAAAGAAAGATT	172	40,000	0	<0.2
Off Target 5	AGAAATGAAAGAAAGAGA	150	40,000	0	<0.2
Off Target 6	GGTGGTGAAAGAAAGAGA	165	40,000	0	<0.2
Off Target 7	AGGACTGAAAGAAAGAGT	154	40,000	0	<0.2
Total off‐target			280,000	0	<0.2

*Note*: Genomic DNA from four different mice post‐NP treatment was subject to deep sequencing analysis at these loci of partial homology. The size of the region sequenced around each site, amplicons sequenced, and the number of amplicons with modified sequences are listed.

**TABLE 4:** Seven gene loci in the mouse genome with partial homology to the 18 bp γPNA target site in beta‐globin intron 2 were previously identified,^2^ with the sequences as indicated

Site name	Sequences of partial homology (5′–3′)	Size of region sequenced	Alleles analyzed	Number modified	Frequency (%)
Off Target 1	AGCCCTGAAAGAAAGAGA	111	600	0	<0.2
Off Target 2	GAACCTGAAAGAAAGAGA	101	600	0	<0.2
Off Target 3	CACCCTGAAAGAAAGAAG	115	600	0	<0.2
Off Target 4	AAGCCTGAAAGAAAGATT	172	600	0	<0.2
Off Target 5	AGAAATGAAAGAAAGAGA	150	600	0	<0.2
Off Target 6	GGTGGTGAAAGAAAGAGA	165	600	0	<0.2
Off Target 7	AGGACTGAAAGAAAGAGT	154	600	0	<0.2
Total off‐target			4200	0	<0.2

*Note*: Genomic DNA from e5.5 blastocysts post‐NP treatment was subjected to deep sequencing analysis at the loci of partial homology indicated in the table. Three embryos were sequenced for each off‐target site. Blastocysts have approximately 100 cells; for homozygous eGFP mice there are approximately 200 alleles per blastocyst. The size of the region sequenced around each site, approximate number of alleles sequenced, and the number of amplicons with modified sequences are listed.

## 3 DISCUSSION

Here, we have developed a technique to use polymeric NPs loaded with PNAs and donor DNA to achieve site‐specific nonenzymatic genome editing in fertilized mouse embryos. As NPs are simply pipetted into medium with embryos, this technique represents an easier alternative to CRISPR/Cas9 microinjection. We found that PNA/DNA NP treatment did not cause detectable cytotoxicity and allowed for normal cell morphology, cellular division, and zona hatching at the correct developmental stage. Embryos reimplanted at the one and two‐cell stage of development also exhibited normal growth with minimal embryo loss due to treatment, suggesting that PNA/DNA NP treatment may be a gentler alternative to CRISPR/Cas9 microinjections, avoiding the significant embryo loss seen with microinjection. Using dye‐loaded NPs, we observed consistent uptake and distribution in embryos, and we found that labeled PNAs were widely distributed across the embryos, including in the nuclei, which persisted up to at least 5 days.

Analysis of gene editing revealed that the average level of editing achieved at the blastocyst stage at the dose of 500 μg ml^−1^ is higher than the level of editing seen in e17.5 or p21 pups derived from the one‐cell and two‐cell reimplantations, which may indicate that reimplantation at the earlier stages does not allow for the continued editing that could occur when the embryos are allowed to develop into blastocysts in culture. This is also consistent with results of reimplantation studies at different developmental timepoints, as we found higher levels of genotypic and phenotypic editing in mice reimplanted at the two‐cell stage than the single‐cell stage.

In addition, during early embryonic development, the genome is transcriptionally silenced and the embryo contains all necessary signals for early cell divisions without any genomic transcription.^30^ The embryo then undergoes the maternal‐to‐zygotic transition (MZT) when maternal mRNAs are degraded and the genome becomes transcriptionally active.^30^ MZT occurs after the two‐cell stage in mice and after the four‐cell stage in humans.^30^ Prior to the MZT, genomic DNA exists in a repressed state that is incompatible with transcription.^31^ PNA‐mediated editing acts through binding and formation of a PNA/DNA/PNA triplex that induces homologous recombination and genome editing.^32^ Since DNA methylation and tight chromatin organization contributes to transcriptional repression in the early embryo,^31^ this chromatin state could reduce the ability of PNA to bind and induce editing. Once the MZT occurs, the chromatin environment may be more conducive to editing, which is consistent with the higher level of editing observed in the blastocysts maintained in culture. Reimplantation of human embryos typically occurs at the blastocyst stage; however, implantation of mouse embryos is most typically done at the single‐cell stage. If PNA editing is limited prior to the MZT, this could represent a significant barrier to the use of this technology in the manipulation of livestock and in the creation of transgenic mouse models, and the mechanism and timing of PNA editing within embryos need to be further elucidated in future studies.

The reimplantation process itself may also encourage NP and/or PNA clearance from the embryo, due to the differences in the in vivo environment of the oviduct and uterus. PNA persists within the embryo in vitro for at least 5 days (Figure 2). In contrast, in vivo, oviductal fluid nourishes the early embryo as it journeys to the uterus; within the uterus the embryo is constantly bathed in endometrial secretions.^33^ These factors may lead to increased fluid exchange in vivo—over what occurs in culture conditions—which could lead to clearing of PNA and/or NPs not observed in vitro. These differences could explain the decreased editing in reimplanted pups compared to editing seen in blastocysts in vitro. These factors may also contribute to the mosaic phenotype observed in the treated mice and should be further investigated in future studies.

Encouragingly, the PNA/DNA NP treatment resulted in undetectable off‐target genomic effects by both targeted and untargeted analyses. These findings are similar to embryo editing by CRISPR microinjection.^34^ However, the application of this approach to human embryos will require substantial further study to determine safety and efficacy. Although pursuing research on embryo editing with implications to treating humans is important, due to safety concerns primarily around potential off‐target effects and mosaicism, therapeutic gene editing of human embryos is unlikely to occur until many future studies on safety and efficacy have been performed. While initial analysis on off‐target events in our PNA/DNA NP treatment are encouraging, it is difficult to ascertain within whole genome sequencing if an individual detected change from the reference genome is naturally occurring or a result of treatment. This makes it extremely difficult to detect a small number of off‐targets events in unexpected loci that could potentially have deleterious effects on an organism. Off‐target analysis techniques likely need to advance before human embryo editing could be considered. Mosaicism is also a concern with human embryo editing, given that mosaicism would make it difficult to assess whether editing of embryos was successful clinically, leading to increased risk of an offspring later developing the genetic disease despite treatment, and makes off‐target effects more difficult to assess. Therefore, although initial editing results for PNA/DNA NP treatment is encouraging, future work should address strategies to reduce mosaicism. Using CRISPR co‐injected with sperm, Ma et al.^4^ were able to reduce mosaicism present in their edited human embryos. A similar study should be conducted with PNA/DNA technology to investigate whether similar results would be achieved.

Ethical questions also surround the use of gene editing in human embryos. Currently, access to assisted reproductive technologies (ART) is not equitable. Few states in the United States mandate health insurance coverage for ART services, including preimplantation genetic diagnosis for couples who have or are carriers for genetic diseases.^35^, ^36^ Similarly, if available, access to embryo editing services in IVF clinics is unlikely to be accessible to all patients regardless of socioeconomic status. Additionally, editing embryos for disease amelioration may open the way to embryo editing for desired superficial traits, such as height or eye color, which would be of potential risk to a developing embryo without clear benefit. Currently nonmedical sex selection is allowed in some IVF clinics for the sole purpose of family balancing, which more often results in male births.^35^ Although perhaps unlikely, it is not impossible that embryo editing could be used in the future for the selection of superficial traits for nonmedical reasons, which would be of significant ethical concern. However, concerns over future nonmedical uses should not limit the development of embryonic gene editing for clinical applications for genetic diseases representing a significant morbidity or mortality risk. Due to multiple ethical and safety concerns surrounding embryo editing in humans, the near future applications of this technology will likely be limited to livestock and research animal manipulation.

The work reported here serves as an initial proof of principle for embryonic gene editing using peptide nucleic acids and donor DNA oligonucleotides encapsulated in PLGA NPs. It may provide a basis for a new approach for genetic manipulation of livestock and research animals at the embryo stage and as a potential future strategy for the treatment of human embryos.

## 4 METHODS

### 4.1 Materials

PLGA, 50:50 ester‐terminated, 0.55–0.75 g dl^−1^ was obtained from LACTEL Absorbable Polymers (Birmingham, AL). C6, polyvinyl alcohol (average molecular weight 30,000–70,000), and dichloromethane were obtained from Sigma Aldrich (St Louis, MO).

### 4.2 Mouse models

All animal use was in accordance with the guidelines of the Animal Care and Use Committee of Yale University and conformed to the recommendations in the Guide for the Care and Use of Laboratory Animals (Institute of Laboratory Animal Resources, National Research Council, National Academy of Sciences, 1996). The 654‐eGFP mice were obtained from Jackson Laboratories (strain number 027617). The 654‐eGFP mice used in the study were homozygous with two copies of a GFP‐beta globin fusion transgene with a IVS2‐654 (C to T) mutation causing an aberrantly expressed intron. Successful gene editing results in expression of a functional GFP mRNA transcript and eGFP fluorescence.^22^

### 4.3 Superovulation and embryo harvest

To obtain single‐cell fertilized embryos, 5‐ to 8‐week‐old female mice were superovulated by intraperitoneal (IP) injection with 5 IU pregnant mare's serum gonadotropin (PMSG) (Peptides International, #HOR‐272). After 48 h, 5 IU of human chorionic gonadotropin (HCG) was administered via IP injection (Millipore Sigma, #869031‐100UG). The hormone treated females were mated with stud mice overnight and examined for sperm plugs the next morning; plugged females were euthanized. Their oviducts were dissected and placed into a petri dish containing light mineral oil and a 50 μl droplet of hyaluronidase solution (Irvine Scientific #90101) and three 50 μl droplets of Embryomax M2 media (Sigma, #M7167). The ampulla of the oviduct was torn using a 31G needle to release the fertilized embryos. Harvested embryos were immediately transferred to a drop of hyaluronidase solution to digest off the cumulus cells for 30 s. Embryos were washed with fresh hyaluronidase‐free M2 media three times and then transferred to 72‐well 10 μl tissue culture dish (1–4 embryos per well) containing KSOM media (EMD Millipore, #MR‐106‐D) preequilibrated for an hour in a 37°C incubator with 5% CO_2_. The 72 well plate containing embryos was covered in 7 ml of light mineral oil (Sigma, #M5310) prior to transfer to the 37°C incubator.^1^, ^37^

### 4.4 Oligonucleotides

The PNA oligomers were synthesized on MBHA (4‐methylbenzhydrylamine) resin according to standard procedures of Boc chemistry using monomers obtained from ASM Research Chemicals (Hannover, Germany).^38^, ^39^ A Kaiser test was performed at each step to measure complete coupling and double coupling was performed if it was required. The oligomers were cleaved from the resin using an *m*‐cresol/thioanisole/TFMSA/TFA (1:1:2:6) cocktail, and the resulting mixtures were precipitated with ethyl ether, purified by reversed phase‐high‐performance liquid chromatography (acetonitrile:water) and characterized with a matrix‐assisted laser desorption/ionization time‐of‐flight mass spectrometer.^40^ The sequence of γPNA used in this study is H‐KKK‐JTTTJTTTJTJT‐OOO‐TCTCTTTCTTTCAGGGCA‐KKK‐NH_2_. Underlined indicates γPNA residues; K, lysine; J, pseudoisocytosine; O, 8‐amino‐2,6,10‐trioxaoctanoic acid linkers connecting the Hoogsteen and Watson‐Crick domains of the tcPNA. During PNA synthesis, the fluorescent dye 5‐carboxytetramethylrhodamine (TAMRA; Biotium) was conjugated to the N‐terminus of a fraction of the synthesized PNA prior to cleavage with *m*‐cresol/thioanisole/TFMSA/TFA. The single‐stranded donor DNA oligomer was obtained from Midland Certified Reagent Company Inc. (Midland Texas) and prepared by standard DNA synthesis except for the inclusion of three phosphorothioate internucleoside linkages at each end to protect against nuclease degradation. The 60 bp donor DNA matches positions 624–684 in beta‐globin intron 2, with the correcting IVS2‐654 nucleotide underlined: 5′‐AAAGAATAACAGTGATAATTTCTGGGTTAAGGCAATAGCAATATCTCTGCATATAAATAT‐3′.

### 4.5 Nanoparticle fabrication

NPs containing C6 were formulated using a single‐emulsion solvent evaporation technique.^12^ C6 was added to the polymer solution at a 0.2% wt:wt dye: polymer ratio. C6 and 80 mg of PLGA were dissolved in 1 ml of dichloromethane (DCM) overnight. This mixture was added dropwise to 2 ml of 5% aqueous polyvinyl alcohol (PVA), then ultrasonicated (3 × 10 s, 38% amplitude) to formulate a single emulsion. This mixture was poured into 20 ml of 0.3% aqueous PVA and stirred for 3 h at room temperature. NPs were then thoroughly washed with 20 ml water (3×) and further collected each time by centrifugation (16,100 × *g* for 10 min at 4°C). NPs were resuspended in water, flash frozen in liquid nitrogen, and then lyophilized. NPs were stored at −20°C after lyophilization.^8^

PNA/DNA PLGA NPs were formulated using a double‐emulsion solvent evaporation technique modified to encapsulate PNA and DNA oligomers.^8^, ^20^ PNAs and donor DNAs were dissolved in DNAse‐free water at a 1 mM concentration. All NP batches used 2 nmole mg^−1^ of γPNA and 1 nmole mg^−1^ of donor DNA. The encapsulant was added dropwise to a polymer solution containing 80 mg PLGA dissolved in dichloromethane (1 ml), then ultrasonicated (3 × 10 s) to formulate the first emulsion. To form the second emulsion, the first emulsion was added slowly dropwise to 2 ml of 5% aqueous polyvinyl alcohol and then ultrasonicated (3 × 10 s). This mixture was finally poured into 20 ml of 0.3% aqueous polyvinyl alcohol and stirred for 3 h at room temperature. NPs were then thoroughly washed with 20 ml water (3×) and further collected each time by centrifugation (16,100 × *g* for 10 min at 4°C). NPs were resuspended in water with cryoprotectant (trehalose, mg:mg), flash frozen in liquid nitrogen, and then lyophilized. Nanoparticles were stored at −20°C after lyophilization.^8^ Dynamic light scattering (DLS) was performed to measure the NPs size (hydrodynamic diameter) and surface charge (zeta potential) using a Malvern Nano‐ZS (Malvern Instruments, UK) (Table S1). To perform scanning electron microscopy (SEM), PNA/DNA nanoparticles at 0.25 mg/mL were added to SEM imaging discs, dried for 12–24 h, coated with carbon, then imaged using a Hitachi SU7000 machine (Hitachi, Japan).

### 4.6 Embryo treatment

Embryos were allowed to equilibrate in the KSOM medium within the cell incubator for an hour prior to NP treatment. NPs were resuspended in KSOM prior to NP administration. Embryos were treated with a range of NP concentrations from 0 to 500 μg ml^−1^. One hour after NP treatment, embryos were washed three times and then transferred to a well containing fresh KSOM medium. Prior to imaging, some embryos were stained with Hoescht 33342 to visualize the nuclei. Imaging of live embryos was performed using an EVOS FL Cell Imaging System (Thermo Fischer Scientific, Waltham, MA, USA).

### 4.7 Isolation of gDNA from blastocysts

Blastocyst lysis buffer was made as follows for 10 ml: 500 μl 1 M Tris, 20 μl 0.5 M EDTA, 500 μl of 10% Tween‐20, and 9 ml dH_2_O. Blastocyst lysis buffer has the final concentration: 50 mM Tris pH 8.8, 1 mM EDTA pH 8.0, and 0.5% Tween‐20. Proteinase K was added prior to use: 1.5 μl of proteinase K (20 mg ml^−1^) in 50 μl of lysis buffer. One blastocyst was incubated in 10 μl of lysis buffer at 55°C overnight. In the morning the sample was heat inactivated at 95°C for 15 min and then cooled to room temperature prior to use.

### 4.8 Whole genome amplification

A volume of 2.5 μl of gDNA from the blastocyst digestion as described above was used for whole genome amplification (Lucigen, Sygnis TruePrime WGA Kit #SYG380100 or Expedeon, Trueprime WGA Kit #380100). Kit instructions were as follows: 2.5 μl of buffer D was added to each gDNA sample and allowed to incubate for 3 min to denature the gDNA. The 2.5 μl of buffer N was then added to neutralize the denaturation reaction. Amplification mix was then added as follows: 26.8 μl of dH_2_O, 5 μl reaction buffer, 5 μl dNTPs, 5 μl enzyme 1, and 0.7 μl enzyme 2. Samples were then incubated at 30°C for 6 h then heat inactivated at 65°C for 10 min. Samples were purified using MicroBio‐Spin P‐30 Tris Chromatography columns (BioRad, Hercules, CA). Columns were inverted to resuspend the gel matrix, then centrifuged at 1000 × *g* for 2 min to remove the matrix. Columns were placed in 1.5 ml microcentrifuge tubes and 20 μl of WGA sample was applied to the center of the column. Sample was centrifuged for 4 min at 1000 × *g*. Samples were then placed in −20°C freezer until further use. To check the fidelity of the WGA, a standard curve was constructed from known amounts of gDNA (Figure S3) and the template appears to amplify with high fidelity (*R* ^2^ = 0.9996).

### 4.9 Droplet digital PCR

A volume of 1–10 μl of the whole genome amplified gDNA or gDNA from reimplanted mice was used for droplet digital PCR. PCR reactions were set up as followed: 11 μl 2× ddPCR™ supermix for probes (no dUTP) (Bio‐Rad, Hercules, CA), 0.2 μl forward primer (100 μM), 0.2 μl reverse primer (100 μM), 0.053 μl eGFP/β‐thal probe (100 μM), 0.053 μl wild‐type probe (100 μM) (Integrated DNA Technologies, Coralville, IA), 0.5 μl EcoR1, 10 μl gDNA and dH_2_O. Droplets were generated using the Automated Droplet Generator (AutoDG™) (Bio‐Rad). Thermocycling conditions were as follows: 95°C 10 min (94°C 30 s, 55.3°C 5 min—ramp 2°C/s) × 40 cycles, 98°C 10 min, hold at 4°C. Droplets were allowed to rest at 4°C for at least 30 min after cycling and were then read using the QX200™ Droplet Reader (Bio‐Rad). Data were analyzed using QuantaSoft™ software. The primers used for ddPCR were as follows: forward: 5′‐ACCATTCTAAAGAATAACAGTGA‐3′, reverse: 5′‐CCTCTTACATCAGTTACAATTT‐3′. The probes used for ddPCR were as follows: wild‐type (FAM): 5′‐TGGGTTAAGGCAATAGCAA, eGFP/β‐thal (HEX): 5′‐TCTGGGTTAAGGTAATAGCAAT.

### 4.10 Deep sequencing

For deep sequencing, PCR reactions were performed with high fidelity TAQ polymerase (Invitrogen; Carlsbad, CA). Each PCR tube consisted of 28.2 μl dH_2_O, 10 μl genomic DNA sample, 5 μl 10× HiFi Buffer, 3 μl 50 mM MgCl_2_, 1 μl dNTP, 1 μl each of forward and reverse primer, 0.8 μl High Fidelity Platinum Taq Polymerase and 10 μl gDNA. Thermocycler conditions were as follows: 94°C 2 min (94°C 30 s, 55°C 45 s, 68°C 1 min) × 40 cycles, 68°C 1 min, hold at 4°C. PCR products were purified using the QIAquick PCR Purification Kit (Qiagen; Hilden, Germany). PCR products were prepared by end‐repair and adapter ligation according to Illumina protocols (San Diego, CA), and samples were sequenced by the Illumina NovaSeq with 75 paired‐end reads at the Yale Center for Genome Analysis. Samples were analyzed using Basepair (basepairtech.com). To study off‐target effects, we looked for the presence of nucleotide changes within the off‐target amplicons. The primers used for beta−globin intron 2 were as follows^9^: forward primer: 5′‐NNN NNN TATCATGCCTCTTTGCACCA; reverse primer: 5′‐NNN NNN AGCAATATGAAACCTCTTACATCA. Each N is a random base added to act as a barcode to track a sample through rounds of PCR amplification. Primers for off‐target sites of partial homology were as follows; forward primer is listed first: Off Target 1 (5′‐NNN NNN AGATAATTATTGCCTCCCACTGC‐3′ and 5′‐NNN NNN AATGGAAGGGCATGCAGTCA‐3′); Off Target 2 (5′‐NNN NNN CCCAATCCTG AATCCTGGCT‐3′ and 5′‐NNN NNN CATACTGATGTCTGTGGCTTGA‐3′); Off Target 3 (5′‐NNN NNN AAGCTCAAACCTACCAGACCA‐3′ and 5′‐NNN NNN AGCTGGAAGCTTCTTCAGTCA‐3′); Off Target 4 (5′‐NNN NNN CCCTCTGTGGACTGAGGAAG‐3′ and 5′‐NNN NNN TGATGAGCTACGGGTATGTGA‐3′); Off Target 5 (5′‐NNN NNN CAAAAAGCCTTAAGCAAACACTC‐3′ and 5′‐NNN NNN TCTCTCCCTCAGCATCTATTCC‐3′); Off Target 6 (5′‐NNN NNN TGTGTTTGTTTATGGATACTTGAGC‐3′ and 5′‐NNN NNN GCATGCACAATAAAGGCACT‐3′); Off Target 7 (5′‐NNN NNN CATGGGAAACAGTCAAAAGAAA‐3′ and 5′‐NNN NNN GTAGGTTTCCCCACAGCTT‐3′).

To analyze off‐targets by deep sequencing in embryos, gDNA was extracted from e5.5 blastocysts and subjected to deep sequencing. Three treated embryos and one untreated embryo were analyzed for each off‐target site. Off‐target values are reported as the sum of modifications in the amplicon across the three treated embryos. Untreated embryos showed no sequence modifications in the amplicons.

To analyze off‐targets by deep sequencing in mice, gDNA was extracted from e17.5 fetuses and subjected to deep sequencing. Samples of various tissues from four different mice were analyzed for each off‐target site. Off‐target modifications are reported as the sum of modifications in the amplicon across the total number of analyzed amplicons for the four treated mice. Tissue from two untreated mice was also analyzed for each off‐target site. Untreated mice showed no modifications.

For deep sequencing data analysis, the variant caller FreeBayes on basepairtech.com was used. To be considered a variant, there had to be at least two observations of that change in the amplicon, and the change had to be present at a frequency of at least 0.002 (set as the minimum alternative fraction) (0.2%). Reads for samples at any one base were capped at 10,000 randomly selected reads. The analysis pipeline was developed by basepairtech.com.

In analysis, it was noted that in the Off Target 3 amplicons, all treated and untreated amplicons of both blastocysts and adult tissues had a SNP at position 39305530 in chromosome 3 when compared to the reference mouse (mm10) genome with a modification rate of ≥99.7% so this was not considered a modification for any of the amplicons as it was not a result of treatment.

### 4.11 Whole genome sequencing

Genomic DNA was harvested from livers of treated or untreated mice that had been reimplanted at the two‐cell stage. The Illumina Nextera DNA Flex Library Prep Kit was used to prepare samples for whole genome sequencing (Illumina; San Diego, CA). Samples were labeled with Nextera DNA CD indexes kit (Illumina). Five sample libraries were then pooled together and sequenced on the Illumina NovaSeq machine, 75 bp paired ends. Samples were sequenced at 30× coverage. Whole genome sequencing data were analyzed using Basepair (basepairtech.com). Samples were aligned to the mouse genome (mm10). To call variants, the variant caller FreeBayes was used. To be considered a variant, there needed to be a count of at least two of the alternative observation and there needed to be a variant alternative at a frequency of at least 0.05 (set as the minimum alternative fraction) (5%). To analyze a base, it needed to be present at a depth of at least 10 reads. For filtering out variants present in either parent, variants that were at the same position number within a chromosome or within 15 base pairs of that position number were discarded.

### 4.12 Reimplantation studies

Embryos were harvested from female donor mice according to previously discussed methods. For embryos reimplanted at the single‐cell stage, embryos were treated for 1 h in 50 μl KSOM‐AA media containing 500 μg ml^−1^ PNA/DNA NPs before transfer to surrogate moms. Embryos implanted at the two‐cell stage were cultured according to above protocols. Only healthy appearing two‐cell embryos were reimplanted. The two‐cell embryos were transferred to a 50 μl droplet of KSOM‐AA just prior to reimplantation. Embryos at either the one‐cell or two‐cell stage were transferred to pseudopregnant recipient females that had been mated with vasectomized males the previous night.

All embryo transfers were performed in accordance with the protocols of the Yale Genome Editing Center. Fetal and postnatal development was observed. Genomic DNA for analysis by ddPCR and sequencing methods was isolated using the Reliaprep™ gDNA Tissue Miniprep System (Promega; Madison, WI).

## AUTHOR CONTRIBUTIONS

**Rachael Putman:** Conceptualization (equal); data curation (equal); formal analysis (equal); methodology (equal); writing – original draft (equal). **Adele Ricciardi:** Conceptualization (equal); data curation (equal); formal analysis (equal); investigation (equal); methodology (equal); writing – original draft (supporting); writing – review and editing (equal). **Kelly Carufe:** Data curation (supporting); investigation (supporting); writing – review and editing (supporting). **Elias Quijano:** Data curation (supporting); writing – review and editing (supporting). **Raman Bahal:** Resources (equal). **Peter Glazer:** Conceptualization (supporting); formal analysis (equal); funding acquisition (equal); project administration (equal); resources (equal); supervision (equal); writing – review and editing (equal). **W. Mark Saltzman:** Conceptualization (supporting); formal analysis (equal); funding acquisition (equal); project administration (equal); supervision (equal); writing – review and editing (equal).

## CONFLICT OF INTEREST

Raman Bahal, Peter M. Glazer, Elias Quijano, Adele S. Ricciardi, Rachael Putman, and W. Mark Saltzman are inventors on patent applications describing nanoparticle‐mediated delivery of triplex‐forming PNAs for gene editing. Raman Bahal is an inventor on a patent application regarding the synthesis of γPNAs. Rachael Putman, Adele S. Ricciardi, Peter M. Glazer, and W. Mark Saltzman are inventors on a patent application regarding embryonic gene editing in vitro.

### PEER REVIEW

The peer review history for this article is available at https://publons.com/publon/10.1002/btm2.10458.

## Supporting information


**Appendix S1:** Supporting Information

## Data Availability

Sequencing data have been deposited in the Sequence Read Archive (https://www.ncbi.nlm.nih.gov/sra) under acquisition number SUB7430845.
