# Supplementary material for: Nanoparticle‐mediated genome editing in single‐cell embryos via peptide nucleic acids
Source: Bioeng Transl Med. 2022 Dec 2;8(3):e10458. doi: 10.1002/btm2.10458 (PMC10189434; doi:10.1002/btm2.10458)
Supplement: Supplementary file 1 — Appendix S1: Supporting Information [file BTM2-8-e10458-s001.docx]

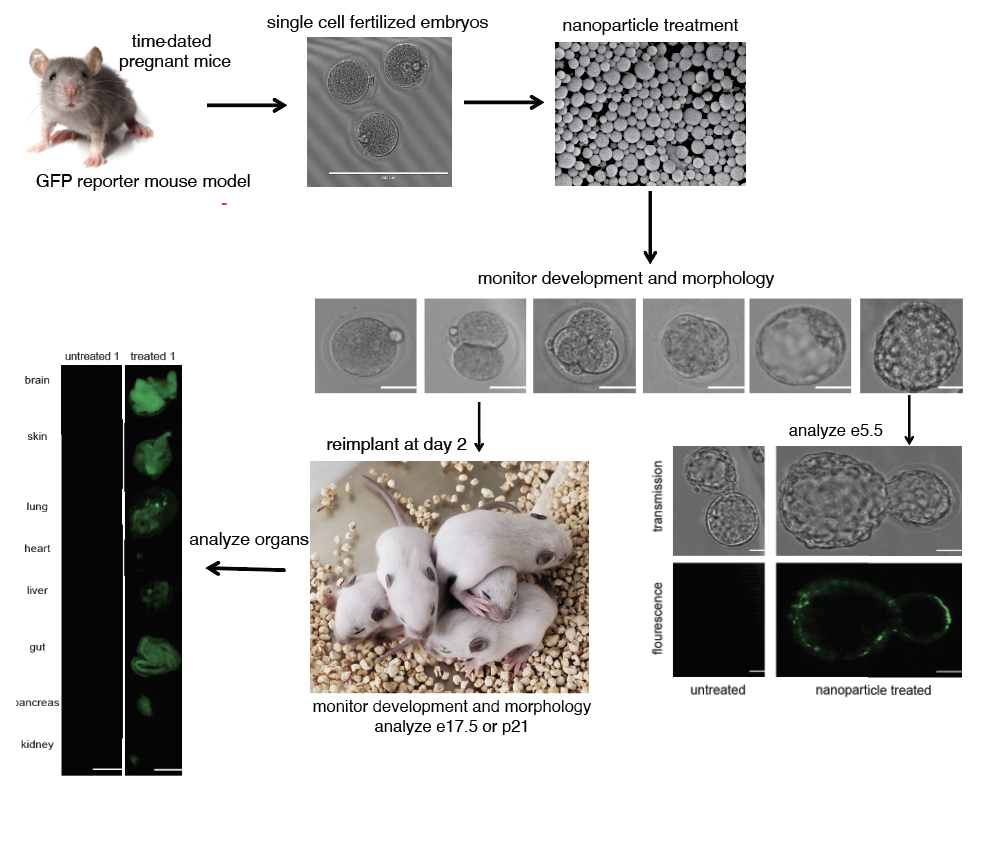
**Graphical Abstract.**

**
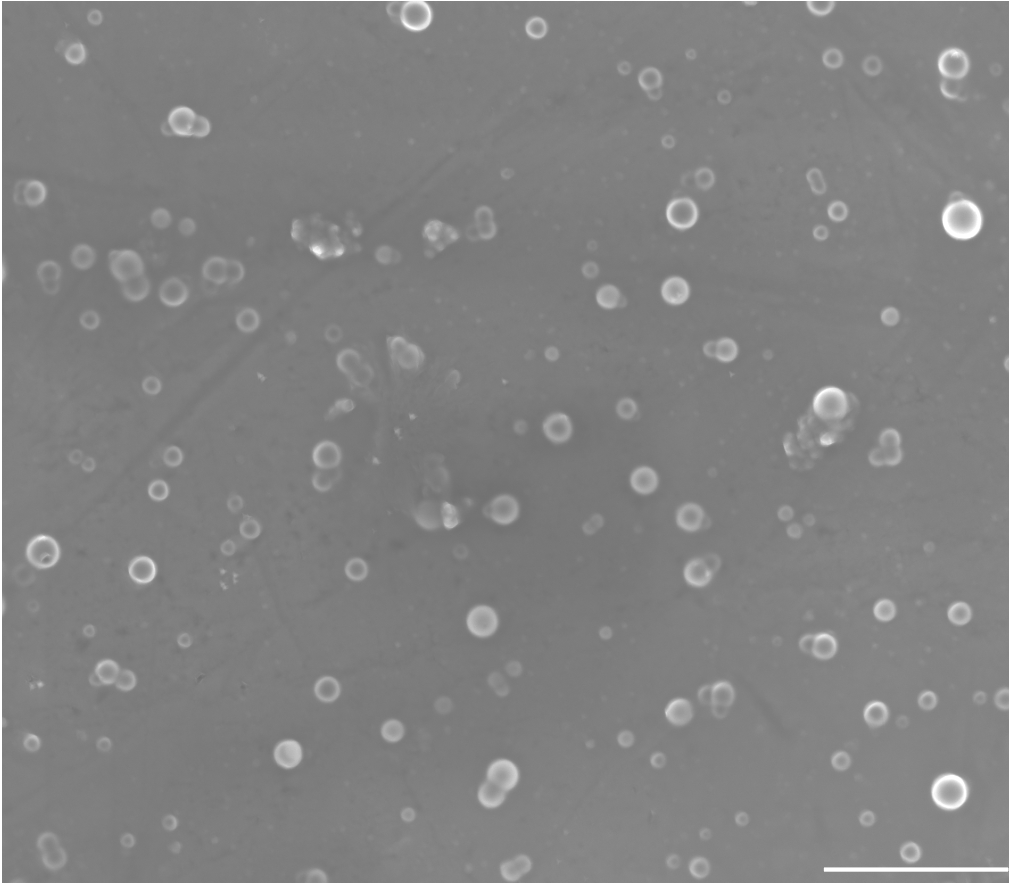
**

**Supplementary Figure 1.** Scanning electron microscopy (SEM) image of PNA/DNA PLGA NPs at 0.25 mg mL^-1^ concentration, scale bar = 2.5 µm.


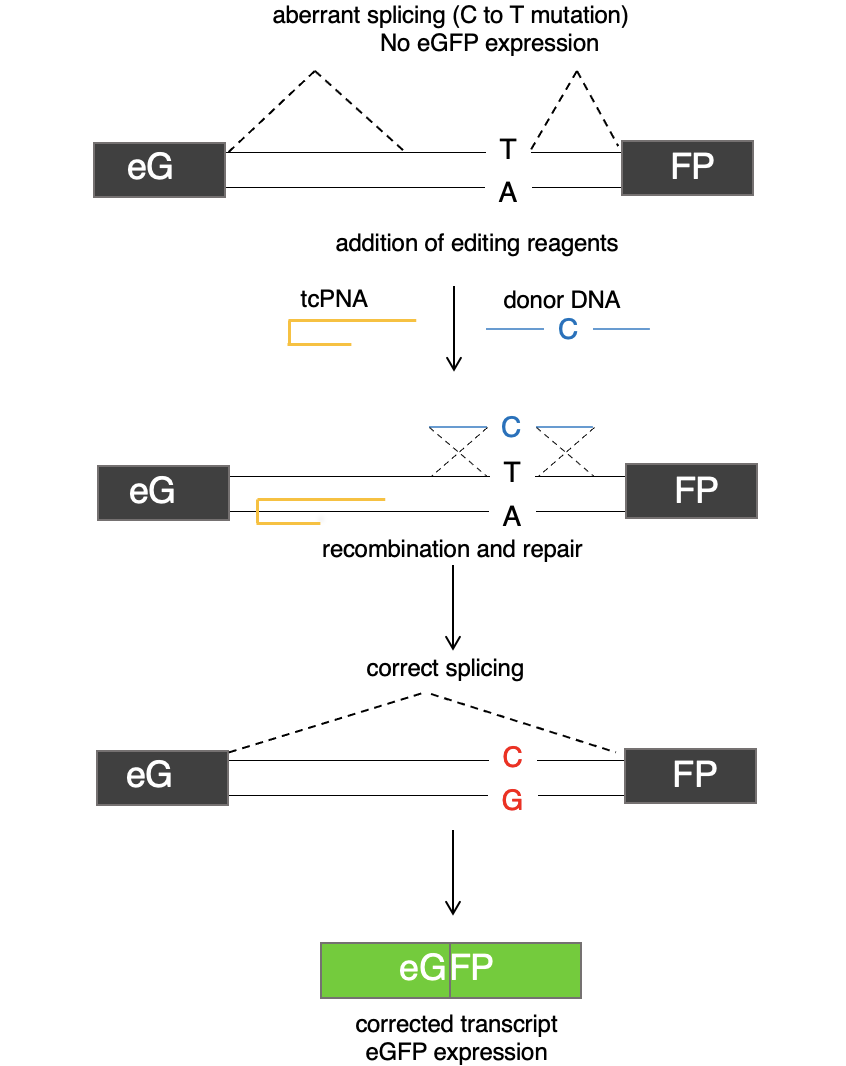


**Supplementary Figure 2**. The mouse model utilized in this study contains a GFP-beta globin fusion transgene with a IVS2-654 C to T mutation in the beta globin-derived intron leading to an aberrantly spliced mRNA. Correction of the mutation allows for functional eGFP expression.
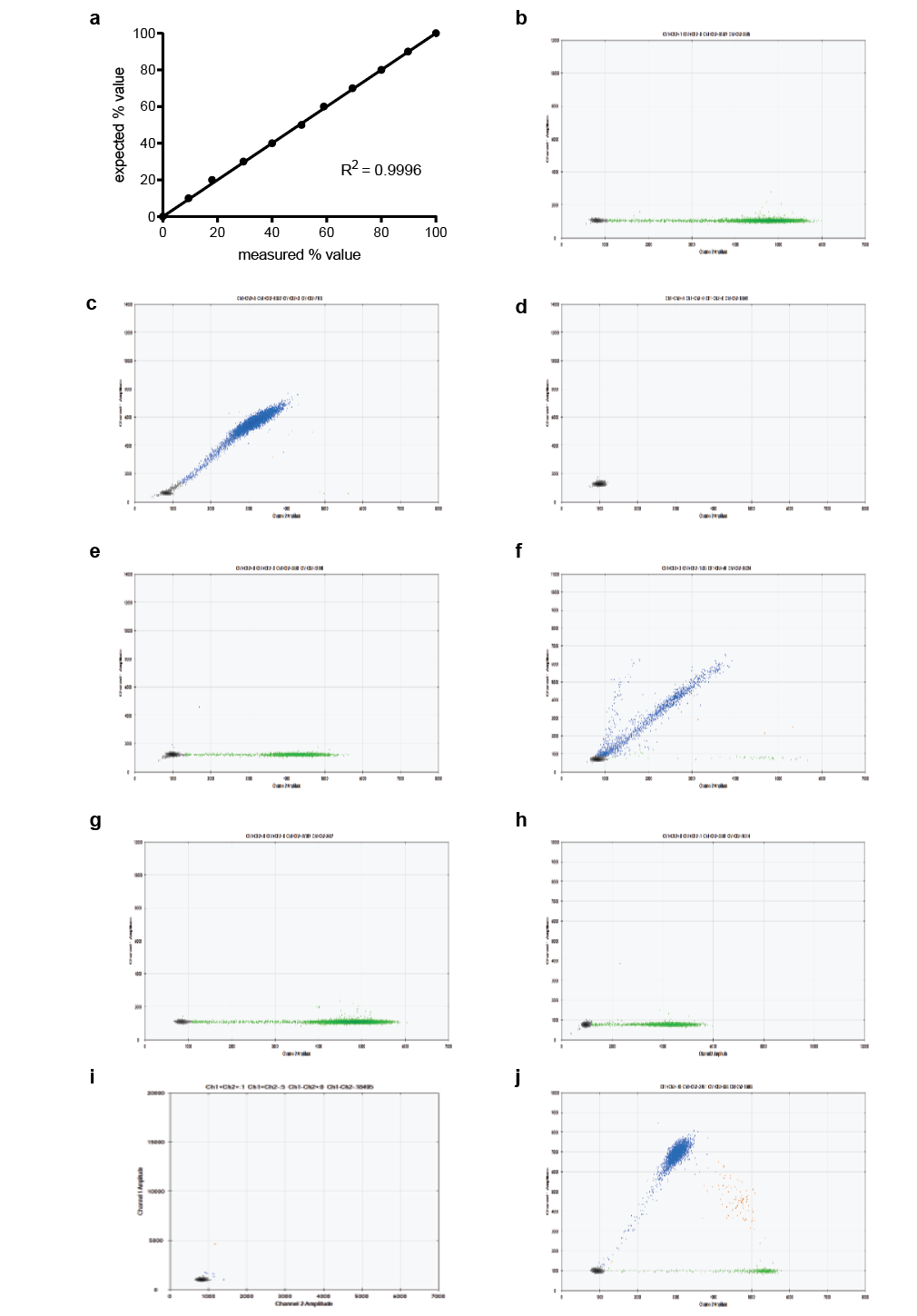
**Supplementary Figure 3**. Validation of whole genome amplification (WGA) and droplet digital PCR (ddPCR) quantification of gene editing. Droplets containing the uncorrected, unedited gDNA sequence amplify as green, droplets with the corrected, edited gDNA amplify as blue, and droplets containing both edited and unedited gDNA amplify as orange. Droplets containing no gDNA are gray. Although this assay was previously validated for investigating gene editing for the 654-eGFP mice^9^, additional controls were performed to ensure WGA did not alter the assay results. **(a)** The measured fractional abundance of the wild-type allele was plotted against the expected fractional abundance of the wild-type allele after whole genome amplification (WGA) was performed on control gDNA. The observed correlation is linear with an R^2^ value of 0.9996. **(b, c, d)** Representative 2D ddPCR plot of gDNA after whole genome amplification are shown for **(b)** untreated eGFP genomic DNA, which is as an unedited control for the assay, **(c)** gDNA from a mouse containing a wild-type human β-globin gene inserted in the region of interest, which is as a positive control for editing in the assay, and (**d)** no template, which is expected to yield no amplification. **(e, f)** representative ddPCR plots showing **(e)** genomic DNA from an untreated blastocyst, and **(f)** genomic DNA from a PNA/DNA NP treated blastocyst. **(g, h, i)** show additional controls for whole genome amplification and the ddPCR assay: **(g)** untreated gDNA incubated with PNA/DNA NPs, **(h)** media from treated samples added to an untreated blastocyst, and **(i)** media containing PNA/DNA NPs but no embryos. For **(i)** incubations with PNA/DNA NPs were performed for 0, 24, 48, and 120 hours. A representative image from 24 hours is shown. **(j)** A representative 2D ddPCR plot is shown for an organ from an eGFP mouse treated as an embryo and analyzed for editing at post-natal day of life 21.


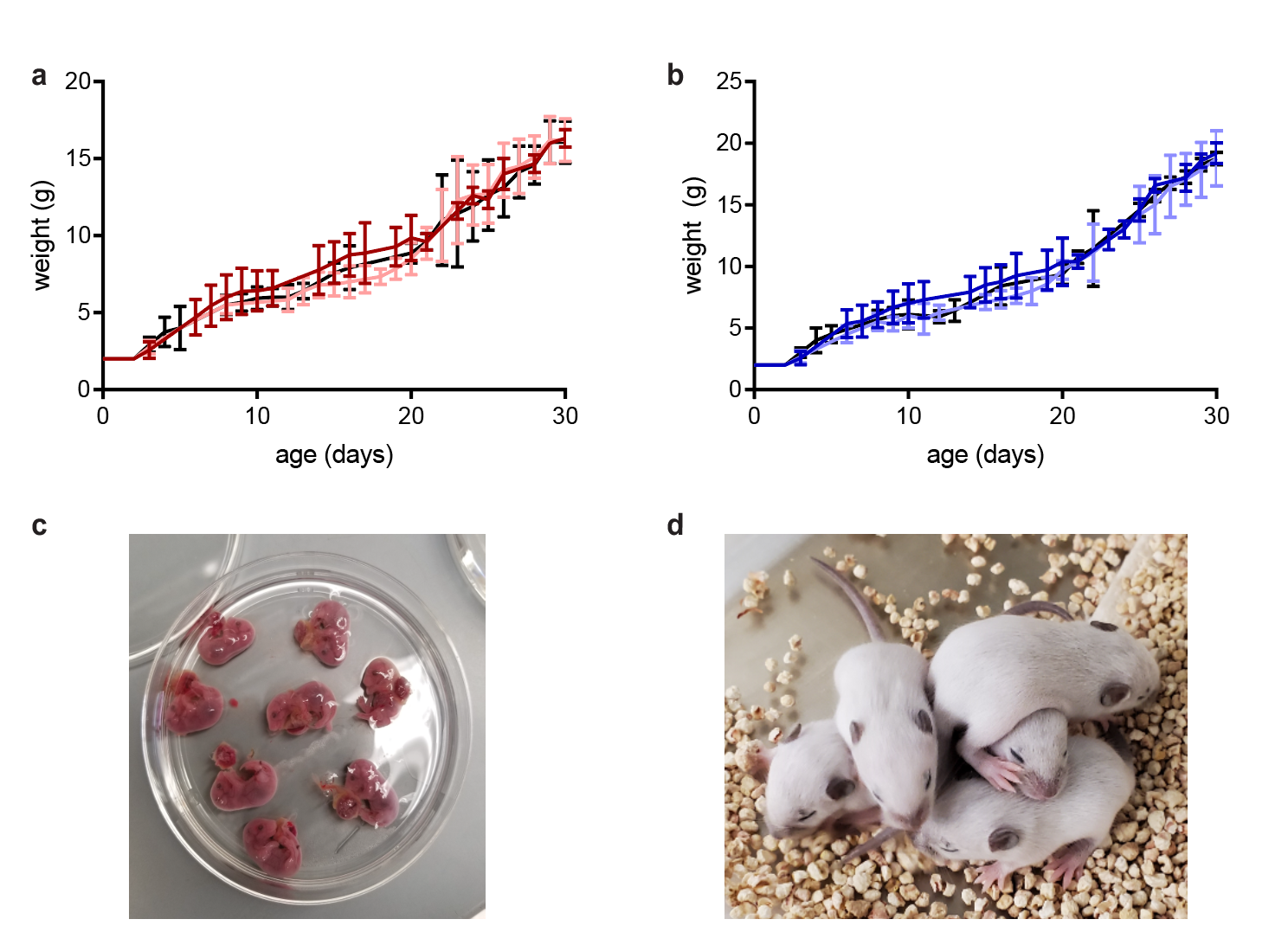
**Supplementary Figure 4. (a, b)** Growth curves from post-natal day of life p1 to p30 are shown for **(a)** females and **(b)** males. Error bars are s.d. 30 untreated female and male mice, 17 female (light pink) and 15 male single-cell reimplanted mice (light blue), and 17 female (dark pink) and 15 male two-cell reimplanted mice (dark blue) were followed for this study. **(c, d)** Images of reimplanted embryos grown to **(c)** e17.5 fetuses and **(d)** postnatal p10 treated mice.


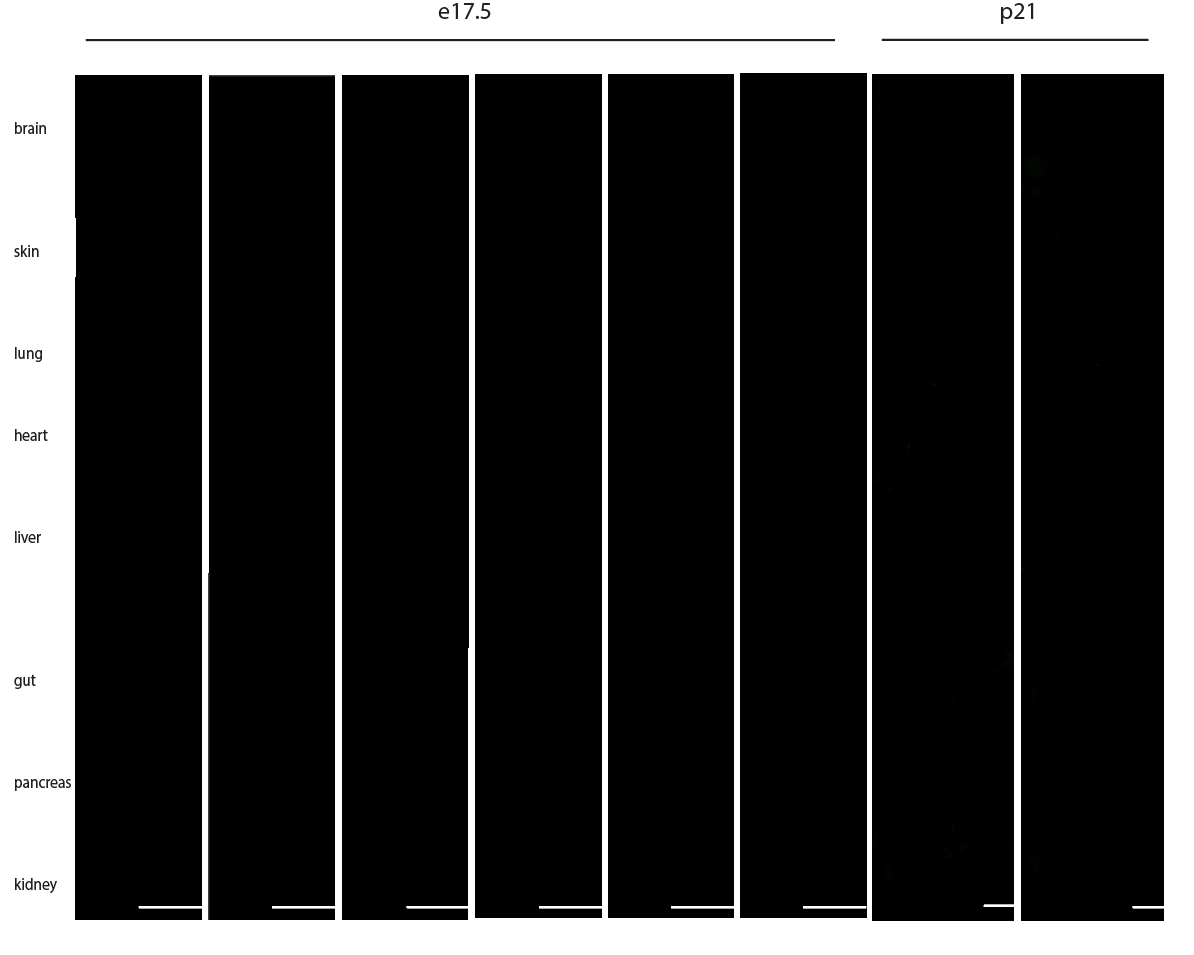
**Supplementary Figure 5**. Absence of green fluorescence indicating lack of phenotypic evidence editing in all untreated controls in fetal e17.5 (left) and adult p21 (right) organs of 654 eGFP mice, scale bars = 2.5 mm.

**
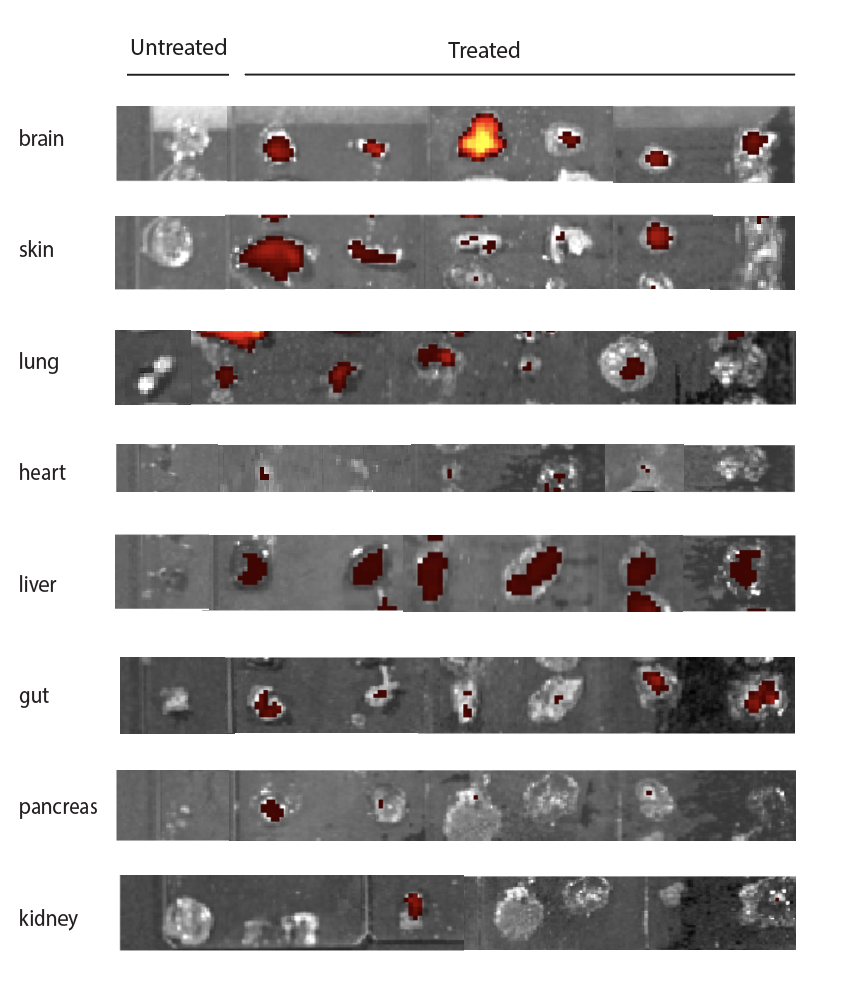
**

**Supplementary Figure 6.** Organs harvested from treated and untreated reimplanted mice at embryonic day of life e17.5 were imaged on IVIS Spectrum In Vivo Imaging System. Organs exhibited different levels of autofluorescence so were taken at settings at which untreated mice showed no fluorescence. Images from one untreated and six treated mice are shown.

**
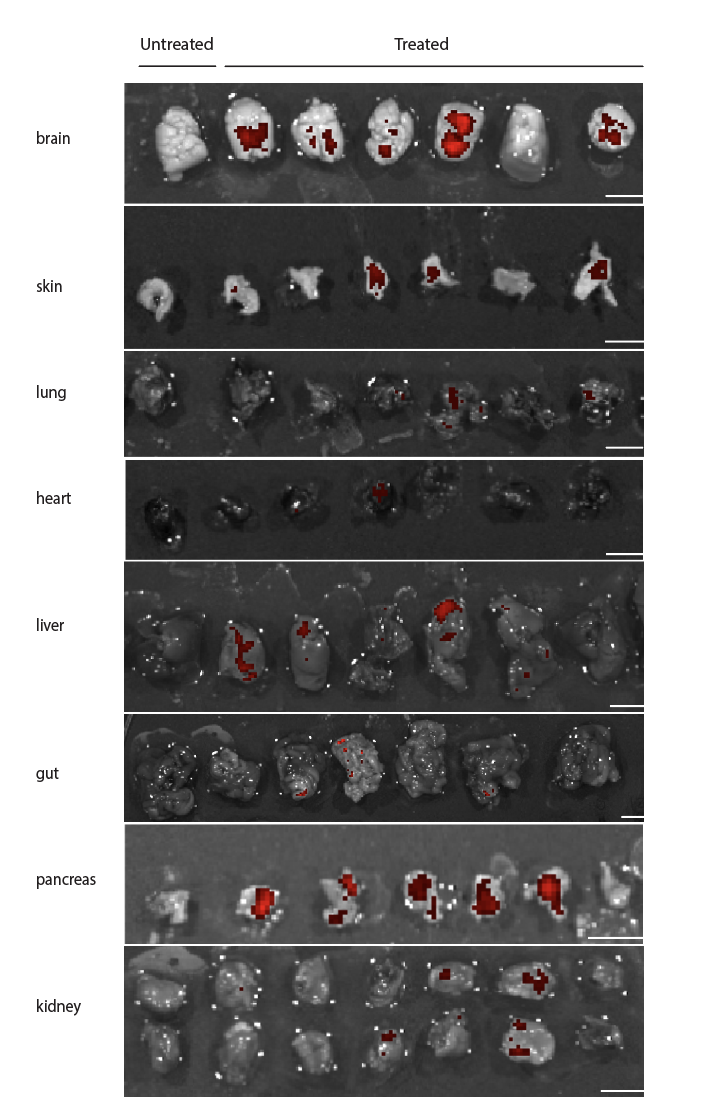
**

**Supplementary Figure 7.** Organs harvested from treated and untreated reimplanted mice at post-natal day of life p21 were imaged on IVIS Spectrum In Vivo Imaging System. Organs exhibited different levels of autofluorescence so were taken at settings at which untreated mice showed no fluorescence. Images from one untreated and six treated mice are shown. Scale bars = 5 mm.


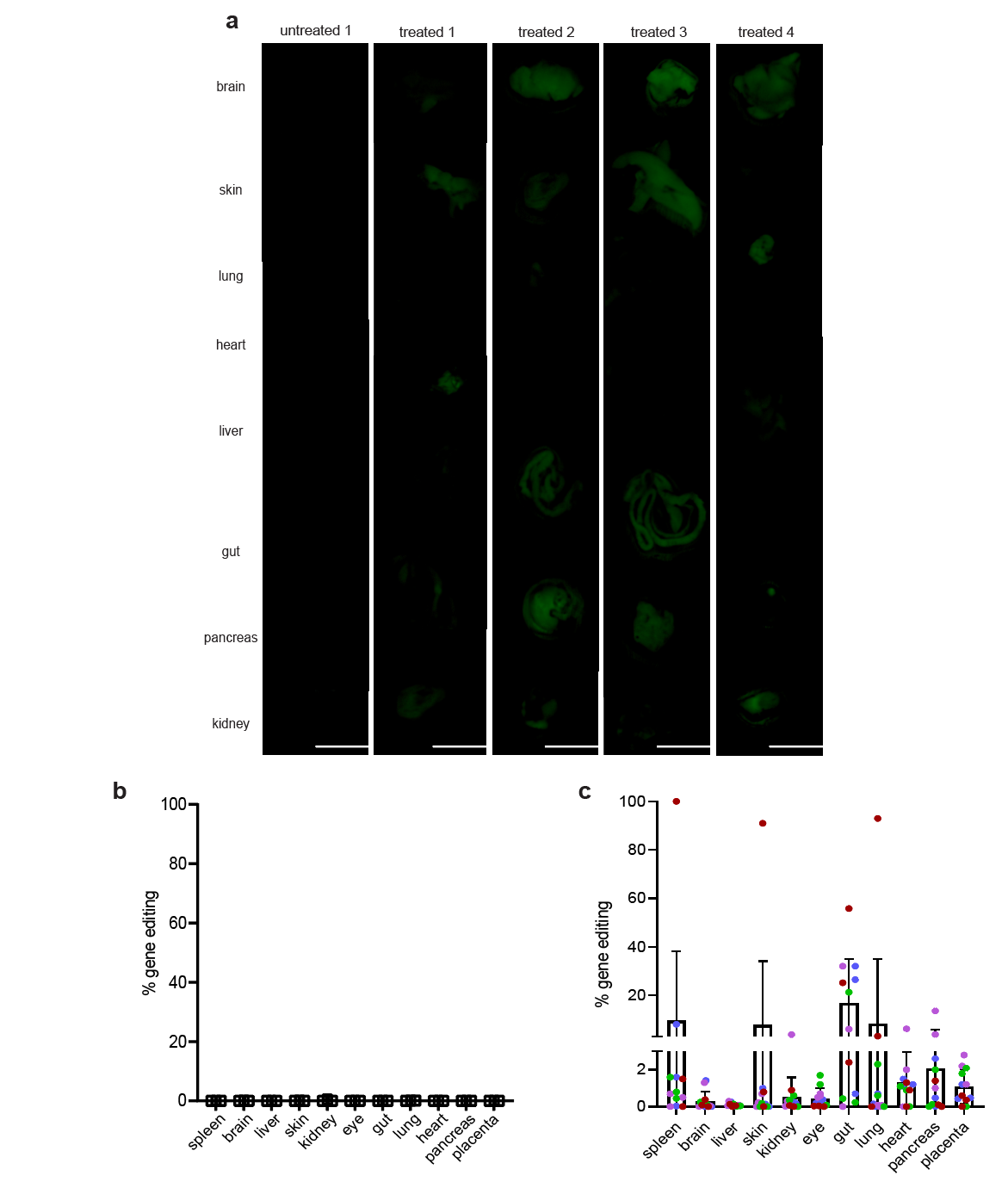


**Supplementary Figure 8**. Gene editing results of reimplantation studies of 654-eGFP embryos performed at the single cell stage. **(a)** eGFP fluorescent signal in tissues of untreated and treated single-cell reimplanted mice. **(b, c)** ddPCR analysis of gene editing for organs of **(b)** untreated and **(c)** treated mice. Percentages from biopsies are shown with mean ± s.e.m, scale bars = 2.5 mm. Three biopsies per mouse organ are plotted. Untreated 1 is plotted on **(b)** and treated 1-4 are plotted on **(c).** Each mouse was assigned its own color in the plots. For **(b)** treated 1 is assigned blue, treated 2 is assigned purple, treated 3 is assigned green, and treated 4 is assigned red.

| Nanoparticle Group | z-avg (nm) | PDI | zeta potential (mV) | Loading (OD mg^-1^ mL ^-1^) |
| --- | --- | --- | --- | --- |
| γPNA/DNA | 239.4 +/- 4.7 | 0.04 +/- 0.01 | -25.6 +/- 6 | 0.46 |
| blank | 336.4 +/- 7.9 | 0.14 +/- 0.02 | -18.5 +/- 0.68 | N/A |
| tamra-PNA | 307.1 +/- 4.9 | 0.10 +/- 0.02 | -21.0 +/- 5.1 | 0.42 |
| coumarin 6 dye | 252.5 +/- 5.1 | 0.11 +/- 0.01 | -25.1 +/- 5.9 | N/A |

**Supplementary Table 1.** Nanoparticles used in the study were characterized by dynamic light scattering (DLS) using a Malvern Nano-ZS. Size (z-avg), polydispersity index values (PDI), surface charge (zeta potential), and nucleic acid loading (loading) are indicated.

| Treatment Group (μg mL^-1^) | Mean % Survival | Number of Embryos |
| --- | --- | --- |
| untreated | 89.9% | 237 |
| vehicle treated | 91.7% | 36 |
| 12.5 | 90.9% | 66 |
| 25 | 88.0% | 92 |
| 50 | 94.4% | 90 |
| 100 | 94.1% | 85 |
| 200 | 97.2% | 36 |
| 300 | 94.5% | 55 |
| 400 | 92.5% | 40 |
| 500 | 88.9% | 477 |

**Supplementary Table 2**. Rates of survival to the 2-cell stage for treated and untreated embryos.

| Treatment Group (μg mL^-1^) | Mean % Survival | Number of Blastocysts |
| --- | --- | --- |
| untreated | 85.7% | 91 |
| vehicle treated | 82.8% | 29 |
| 12.5 | 87.0% | 54 |
| 25 | 81.2% | 69 |
| 50 | 81.6% | 76 |
| 100 | 79.5% | 73 |
| 200 | 82.1% | 28 |
| 300 | 77.8% | 36 |
| 400 | 78.8% | 33 |
| 500 | 84.0% | 94 |

**Supplementary Table 3**. Blastocyst survival rates for treated and untreated embryos.

| Treatment Group (μg mL^-1^) | Mean % Editing | p-value | Number of Blastocysts |
| --- | --- | --- | --- |
| untreated | 0.2% | - | 26 |
| vehicle treated | 0.2% | >0.9999 | 20 |
| 12.5 | 67.4% | <0.0001 | 20 |
| 25 | 68.9% | <0.0001 | 20 |
| 50 | 70.3% | <0.0001 | 20 |
| 100 | 77.6% | <0.0001 | 23 |
| 200 | 80.7% | <0.0001 | 21 |
| 300 | 81.3% | <0.0001 | 20 |
| 400 | 83.8% | <0.0001 | 20 |
| 500 | 94.0% | <0.0001 | 23 |

**Supplementary Table 4**. Blastocyst editing after PNA/DNA treatment at the single cell stage. p-values for treated and vehicle treated groups were found by one-way ANOVA comparison to untreated group.

| **Sample Name** | **Alleles Analyzed** | **Amplicons Analyzed** | **Number modified** | **Frequency** |
| --- | --- | --- | --- | --- |
| treated skin | N/A | 7553051 | 11989403 | 63.0% |
| treated kidney | N/A | 5859106 | 9581762 | 61.1% |
| treated gut | N/A | 7757826 | 3404243 | 43.9% |
| treated heart | N/A | 10511176 | 3111342 | 29.6% |
| treated pancreas | N/A | 8785292 | 3283610 | 37.4% |
| untreated spleen #1 | N/A | 12284090 | 27518 | 0.2% |
| untreated spleen #2 | N/A | 12213373 | 26672 | 0.2% |
| untreated gut #1 | N/A | 12066384 | 27431 | 0.2% |
| untreated gut #2 | N/A | 11629728 | 23508 | 0.2% |
| treated blastocyst #1 | 200 | 5515608 | 7423946 | 74.3% |
| treated blastocyst #2 | 200 | 2110466 | 2536835 | 83.2% |
| treated blastocyst #3 | 200 | 5333075 | 3871122 | 72.6% |
| untreated blastocyst #1 | 200 | 4267239 | 60274 | 1.4% |
| untreated blastocyst #2 | 200 | 11838915 | 288298 | 2.4% |

**Supplementary Table 5.** Deep sequencing was performed at the β-globin locus. The size of the region sequenced was 128 base pairs. Genomic DNA was taken from e5.5 blastocysts that underwent whole genome amplification or from p21 mice that were treated as single-cell embryos and subjected to deep sequencing. Blastocysts have approximately 100 cells; for homozygous eGFP mice there are approximately 200 alleles per blastocyst. The alleles analyzed (blastocysts only), amplicons analyzed, number of amplicons modified, and frequency of gene editing are listed.

| Treatment Group | Mean % Editing | p-value | Samples Analyzed |
| --- | --- | --- | --- |
| post-natal p21 two-cell reimplanted mice | - | - | - |
| Spleen | 2.8 | 0.0093 | 15 |
| Brain | 5.2 | 0.0937 | 15 |
| Liver | 23.5 | 0.0296 | 15 |
| Skin | 9.7 | 0.1345 | 15 |
| Kidney | 10.9 | 0.0283 | 15 |
| Gut | 28.0 | 0.0354 | 15 |
| Lung | 14.3 | 0.0161 | 15 |
| Heart | 1.5 | 0.0001 | 15 |
| Pancreas | 1.7 | 0.0152 | 15 |
| embryonic day of life e17.5 two-cell reimplanted mice | - | - | - |
| Spleen | 9.0 | 0.181 | 9 |
| Brain | 9.6 | 0.0041 | 12 |
| Liver | 12.5 | 0.026 | 12 |
| Skin | 8.6 | 0.0043 | 12 |
| Kidney | `4.7 | 0.0034 | 12 |
| Eye | 22.1 | 0.0291 | 12 |
| Gut | 18.2 | 0.0176 | 12 |
| Stomach | 19.1 | 0.005 | 12 |
| Lung | 21.1 | 0.0141 | 12 |
| Heart | 32.7 | 0.0053 | 12 |
| Pancreas | 36.3 | 0.0005 | 12 |
| embryonic day of life e17.5 single-cell reimplanted mice | - | - | - |
| Spleen | 7.8 | 0.0969 | 9 |
| Brain | 3,3 | 0.3047 | 11 |
| Liver | 4.4 | 0.2902 | 11 |
| Skin | 3.6 | 0.2455 | 11 |
| Kidney | 3.3 | 0.1459 | 11 |
| Eye | 3.4 | 0.2729 | 11 |
| Gut | 10.7 | 0.0212 | 11 |
| Lung | 3.9 | 0.2216 | 11 |
| Heart | 4.2 | 0.1562 | 11 |
| Pancreas | 1.9 | 0.0287 | 11 |
| Placenta | 5.7 | 0.1320 | 11 |

**Supplementary Table 6.** Mice were harvested at either embryonic day of life e17.5 or post-natal day of life p21 after embryonic treatment and reimplantation at the two-cell stage or single-cell stage as indicated. Gene editing was evaluated with droplet digital PCR (ddPCR). Statistical significance of editing was evaluated using one sample t test. Treated samples were compared to hypothetical value of zero percent of completely unedited mice.
